# Role of p*K*_a_ in establishing the crystal structures of six hydrogen-bonded compounds of 4-methyl­quinoline with different isomers of chloro- and nitro-substituted benzoic acids

**DOI:** 10.1107/S2056989021010896

**Published:** 2021-10-26

**Authors:** Hiroyuki Ishida

**Affiliations:** a Department of Chemistry, Faculty of Science, Okayama University, Okayama 700-8530, Japan

**Keywords:** crystal structure, 2-chloro-4-nitro­benzoic acid, 2-chloro-5-nitro­benzoic acid, 2-chloro-6-nitro­benzoic acid, 3-chloro-2-nitro­benzoic acid, 4-chloro-2-nitro­benzoic acid, 5-chloro-2-nitro­benzoic acid, 4-methyl­quinoline, hydrogen bond, disorder, Hirshfeld surface

## Abstract

The structures of the six hydrogen-bonded 1:1 compounds of 4-methyl­quinoline (C_10_H_9_N) with chloro- and nitro-substituted benzoic acids (C_7_H_4_ClNO_4_), namely, 4-methyl­quinolinium 2-chloro-4-nitro­benzoate, C_10_H_10_N^+^·C_7_H_3_ClNO_4_^−^, (I), 4-methyl­quinoline–2-chloro-5-nitro­benzoic acid (1/1), C_10_H_9_N·C_7_H_4_ClNO_4_, (II), 4-methyl­quinolinium 2-chloro-6-nitro­benzoate, C_10_H_9.63_N^0.63+^·C_7_H_3.37_ClNO_4_^0.63−^, (III), 4-methyl­quinolinium 3-chloro-2-nitro­benzoate, C_10_H_9.54_N^0.54+^·C_7_H_3.46_ClNO_4_^0.54−^, (IV), 4-methyl­quinolinium 4-chloro-2-nitro­benzoate, C_10_H_10_N^+^·C_7_H_3_ClNO_4_^−^, (V), and 4-methyl­quinolinium 5-chloro-2-nitro­benzoate, C_10_H_10_N^+^·C_7_H_3_ClNO_4_^−^, have been determined at 185–190 K. In each compound, the acid and base mol­ecules are linked by a short hydrogen bond between a carb­oxy (or carboxyl­ate) O atom and an N atom of the base. The O⋯N distances are 2.5652 (14), 2.556 (3), 2.5485 (13), 2.5364 (13), 2.5568 (13) and 2.5252 (11) Å, respectively, for compounds (I)–(VI). In the hydrogen-bonded acid–base units of (III) and (IV), the H atoms are each disordered over two positions with O site:N site occupancies of 0.37 (3):0.63 (3) and 0.46 (3):0.54 (4), respectively, for (III) and (IV). The H atoms in the hydrogen-bonded units of (I), (V) and (VI) are located at the N-atom site, while the H atom in (II) is located at the O-atom site. In all the crystals of (I)–(VI), π–π stacking inter­actions between the quinoline ring systems and C—H⋯O hydrogen bonds are observed. Similar layer structures are constructed in (IV)–(VI) through these inter­actions together with π–π inter­actions between the benzene rings of the adjacent acid mol­ecules. A short Cl⋯Cl contact and an N—O⋯π inter­action are present in (I), while a C—H⋯Cl hydrogen bond and a π–π inter­action between the benzene ring of the acid mol­ecule and the quinoline ring system in (II), and a C—H⋯π inter­action in (III) are observed. Hirshfeld surfaces for the title compounds mapped over *d*_norm_ and shape index were generated to visualize the weak inter­molecular inter­actions.

## Abstract 2

The structures of the six hydrogen-bonded 1:1 compounds of 4-methyl­quinoline with 2-chloro-4-nitro­benzoic acid, 2-chloro-5-nitro­benzoic acid, 2-chloro-6-nitro­benzoic acid, 3-chloro-2-nitro­benzoic acid, 4-chloro-2-nitro­benzoic acid and 5-chloro-2-nitro­benzoic acid have been determined at 185–190 K. In each crystal, the acid and base mol­ecules are linked by a short hydrogen bond between a carb­oxy/carboxyl­ate O atom and an N atom of the base.

## Chemical context

The properties of hydrogen bonds formed between organic acids and organic bases depend on the p*K*_a_ values of the acids and bases as well as the inter­molecular inter­actions in the crystals. In our ongoing studies of crystal structures for the system of quinoline derivatives–chloro- and nitro-substituted benzoic acids, we have shown that three compounds of quinoline with 3-chloro-2-nitro­benzoic acid, 4-chloro-2-nitro­benzoic acid and 5-chloro-2-nitro­benzoic acid (Gotoh & Ishida, 2009 ▸), and three compounds of 6-methyl­quinoline with 2-chloro-4-nitro­benzoic acid, 3-chloro-2-nitro­benzoic acid and 4-chloro-2-nitro­benzoic acid (Gotoh & Ishida, 2020 ▸) have a short double-well O—H⋯N/O⋯H—N hydrogen bond between the carb­oxy O atom and the aromatic N atom. The Δp*K*_a_ [p*K*_a_(base) – p*K*_a_(acid)] values of these compounds are in the range 2.93–3.38. Although the p*K*_a_ value of 4-methyl­quinoline is 5.66, which is slight larger than quinoline (p*K*_a_ = 4.90) and 6-methyl­quinoline (p*K*_a_ = 5.20), the system of 4-methyl­quinoline–chloro- and nitro-substituted benzoic acids is an attractive candidate for studying short hydrogen bonds and also weak inter­molecular inter­actions. We report here crystal structures of six hydrogen-bonded compounds, namely, 4-methyl­quinolinium 2-chloro-4-nitro­benzoate, (I), 2-chloro-5-nitro­benzoic acid–4-methyl­quinoline, (II), 2-chloro-6-nitro­benzoic acid–4-methyl­quinoline, (III), 3-chloro-2-nitro­benzoic acid–4-methyl­quinoline, (IV), 4-methyl­quinolinium 4-chloro-2-nitro­benzoate, (V), and 4-methyl­quinolinium 5-chloro-2-nitro­benzoate, (VI). The Δp*K*_a_ values are 3.62, 3.44, 4.04, 3.84, 3.69 and 3.80, respectively, for (I)–(VI) (Table 1 ▸). 
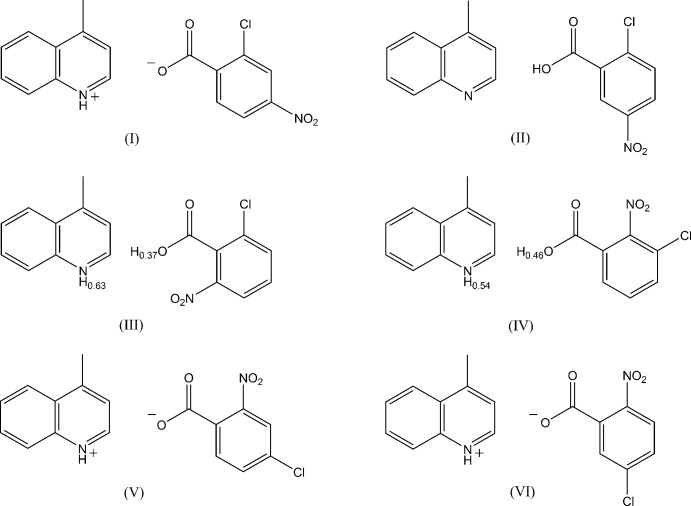


## Structural commentary

**Figure 1:**
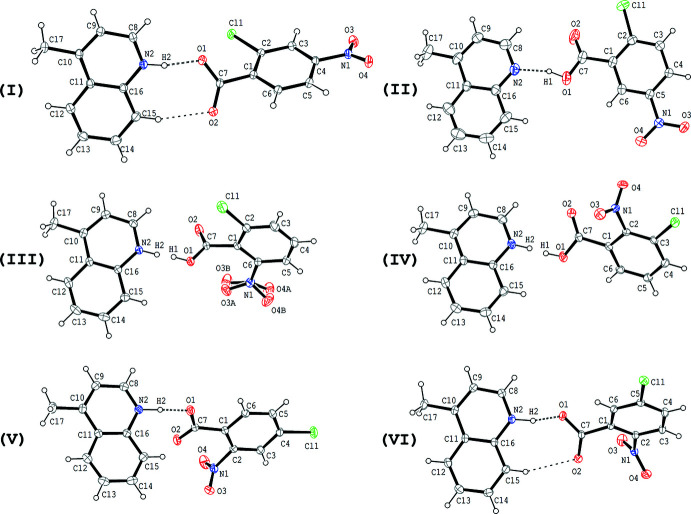
Mol­ecular structures of the title compounds (I)–(VI), showing the atom-numbering scheme. Displacement ellipsoids are drawn at the 50% probability level and H atoms are shown as small spheres of arbitrary radii. In the hydrogen bonds between the carb­oxy O atom and the base N atom of compounds (III) and (IV), the H atoms are each disordered over two positions. The nitro group in (III) is disordered around the N1—C6 bond. Dashed lines in (I), (II), (V) and (VI) indicate the N—H⋯O, O—H⋯N and C—H⋯O hydrogen bonds.

The mol­ecular structures of compounds (I)–(VI) are shown in Fig. 1 ▸. In each compound, the acid and base mol­ecules are linked by a short hydrogen bond between the O atom of the carb­oxy (or carboxyl­ate) group and the N atom of the base with O⋯N distances of 2.5652 (14), 2.556 (3), 2.5485 (13), 2.5364 (13), 2.5568 (13) and 2.5252 (11) Å, respectively, for compounds (1)–(VI) (Tables 2 ▸–7 ▸ ▸ ▸ ▸ ▸). In (III) and (IV), the H atoms in these hydrogen bonds are each disordered over two sites with O site:N site occupancies of 0.37 (3):0.63 (3) and 0.46 (3):0.54 (3), respectively, for (III) and (IV). In (I), (V) and (VI), the H atoms in the hydrogen bonds are located at the N site, while in (II) they are located at the O-atom site. In addition, a weak C—H⋯O hydrogen bond is observed in each of the acid–base units of (I) and (VI) (C15—H15⋯O2; Tables 2 ▸ and 7 ▸). The nitro group in (III) is disordered over two orientations around the N1—C6 bond with occupancies of 0.46 (3) and 0.54 (3).

The dihedral angles made by the benzene C1–C6 ring, the carb­oxy/carboxyl­ate O1/C7/O2 plane and the nitro O3/N1/O4 plane of the acid, and the quinoline N2/C8–C16 ring system of the base in each hydrogen-bonded acid-base unit of (I)–(VI) are summarized in Table 1 ▸, together with those in compounds of other quinoline derivatives with chloro- and nitro-substituted benzoic acids, which contain similar hydrogen-bonded acid-base units (Gotoh & Ishida, 2009 ▸, 2011 ▸, 2019*a* ▸,*b* ▸, 2020 ▸). The H-atom position in the short hydrogen bond and the Δp*K*_a_ value of each compound are also given in Table 1 ▸. In each acid–base unit of compounds of (I) and (III)–(VI), the acid C1–C6 ring and the quinoline N2/C8–C16 ring system are considerably twisted with respect to each other with dihedral angles of 58.90 (4)–69.15 (5)°, which are much larger than those of other compounds. In the acid–base unit of (II), the acid ring and the quinoline ring system are slightly twisted by 13.18 (10)°, which is still larger compared with those of quinoline–2-chloro-5-nitro­benzoic acid [1.92 (4)°] and 6-methyl­quinoline–2-chloro-5-nitro­benzoic acid [2.15 (4)°]. These results suggest that the methyl group substituted to the quinoline ring system at the 4-position has an effect on the mol­ecular packing, which prevents the aromatic rings of the acid and base lying in the same plane in the crystal.

In all the compounds of 3-chloro-2-nitro­benzoic acid and 4-chloro-2-nitro­benzoic acid, the nitro O3/N1/O4 group is approximately perpendicular to the benzene C1–C6 ring with dihedral angles of 74.4 (3)–88.54 (13)°, while in the 2-chloro-6-nitro­benzoic acid mol­ecule of compound (III), where the nitro group and the Cl atom are adjacent to the carb­oxy group, the carb­oxy O1/C7/O2 group is almost perpendicular to the benzene ring with a dihedral angle of 84.53 (16)°. In the compounds of 5-chloro-2-nitro­benzoic acid, the nitro and carb­oxy/carboxyl­ate groups are both twisted by 33.31 (13)–57.13 (11)° out of the benzene ring plane. These large twists are mainly ascribable to intra­molecular steric repulsion between the nitro group and the carb­oxy/carboxyl­ate group.

The correlation between the H-atom position in the short hydrogen bond and the Δp*K*_a_ value is observed for each system of quinoline and 6-methyl­quinoline compounds, while for the title compounds (I)–(VI) this correlation is somewhat low.

## Supra­molecular features

In all the crystals of (I)–(VI), π–π inter­actions between the quinoline ring systems, related by an inversion centre to each other, are observed. The centroid–centroid distances between the quinoline ring systems, namely, *Cg*2⋯*Cg*2, *Cg*2⋯*Cg*3 and *Cg*3⋯*Cg*3, are 3.4323 (7)–3.7751 (8), 3.5878 (7)–3.9304 (9) and 3.7719 (8)–3.9227 (9) Å, respectively, where *Cg*2 and *Cg*3 are the centroids of the N2/C8–C11/C16 and C11–C16 rings of the quinoline ring system, respectively. The base mol­ecules in the crystals of (I) and (II) form dimeric units *via* these π–π inter­actions, while in (III)–(VI) inversion-related base mol­ecules are alternately stacked in column-like structures. On the other hand, π–π inter­actions between the inversion-related acid mol­ecules are only observed in crystals (IV)–(VI); the centroid-centroid distances, *Cg*1⋯*Cg*1, are 3.5702 (7)–3.8602 (6) Å, where *Cg*1 is the centroid of the C1–C6 ring. Detailed supra­molecular features in the crystals formed through these π–π inter­actions combined with other weak inter­molecular inter­actions are described below.

**Figure 2:**
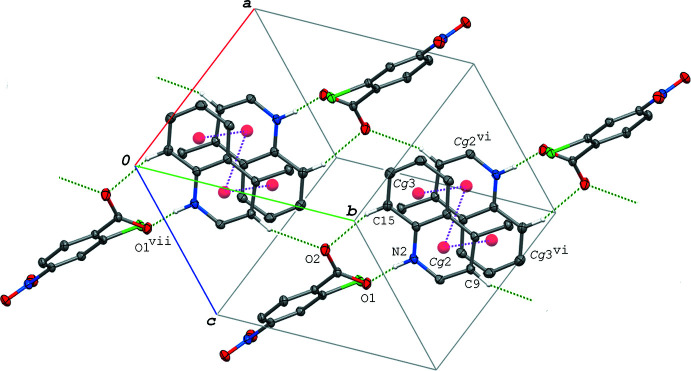
A packing diagram of (I), showing the ribbon structure running along the *b-*axis direction formed *via* the N—H⋯O and C—H⋯O hydrogen bonds (green dashed lines) and π–π inter­actions (magenta dashed lines). H atoms not involved in the hydrogen bonds are omitted for clarity. *Cg*2 and *Cg*3 are the centroids of the N2/C8–C11/C16 and C11–C16 rings, respectively. [Symmetry codes: (vi) −*x* + 1, −*y* + 2, −*z* + 1; (vii) *x*, *y* − 1, *z*.]

**Figure 3:**
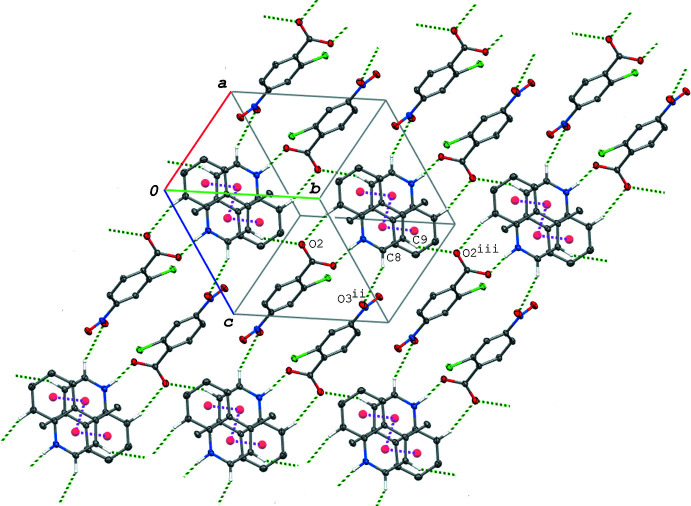
A packing diagram of (I), showing a layer structure parallel to (101) formed *via* the N—H⋯O and C—H⋯O hydrogen bonds (green dashed lines) and π–π inter­actions (magenta dashed lines). H atoms not involved in the hydrogen bonds are omitted for clarity. *Cg*1 is the centroid of the C1–C6 ring. [Symmetry codes: (ii) −*x*, −*y* + 1, −*z* + 2; (iii) *x*, *y* + 1, *z*.]

In the crystal of (I), the hydrogen-bonded acid–base units, which are related by an inversion centre to each other, are linked into a centrosymmetric dimeric unit *via* π–π inter­actions between the quinoline ring systems [*Cg*2⋯*Cg*2^vi^ = 3.7318 (7) Å and *Cg*2⋯*Cg*3^vi^ = 3.5955 (7) Å; symmetry code: (vi) −*x* + 1, −*y* + 2, −*z* + 1]. The dimeric units are further linked *via* a C—H⋯O hydrogen bond (C9—H9⋯O2^iii^; symmetry code as given in Table 2 ▸), forming a ribbon structure propagating along the *b-*axis direction (Fig. 2 ▸). The ribbons are connected into a layer lying parallel to the (101) plane (Fig. 3 ▸) *via* another C—H⋯O hydrogen bond (C8—H8⋯O3^ii^; Table 2 ▸). In the layer, the acid mol­ecules are arranged in an anti­parallel manner with *Cg*1⋯*Cg*1^ii^ = 4.0685 (7) Å. Between the layers, an N—O⋯π inter­action (N1—O3⋯*Cg*3^iv^; Table 2 ▸), a short Cl⋯Cl contact [Cl1⋯Cl1^v^ = 3.3391 (5) Å; symmetry code: (v) −*x* + 1, −*y* + 1, −*z* + 2] and a C—H⋯O hydrogen bond (C6—H6⋯O2^i^; Table 2 ▸) are observed.

**Figure 4:**
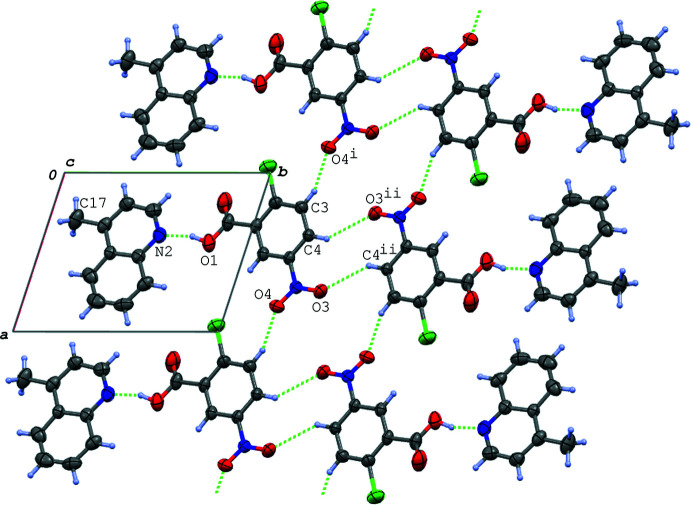
A packing diagram of (II) viewed along the *c* axis, showing the tape structure formed *via* the C—H⋯O hydrogen bonds (green dashed lines). [Symmetry codes: (i) *x* − 1, *y*, *z*; (ii) −*x* + 1, −*y* + 3, −*z* + 1.]

**Figure 5:**
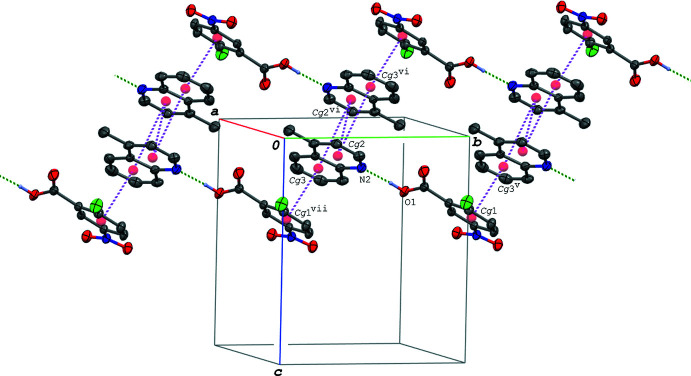
A packing diagram of (II), showing the ribbon structure running along the *b-*axis direction formed *via* the O—H⋯N hydrogen bonds (green dashed lines) and π–π inter­actions (magenta dashed lines). H atoms not involved in the hydrogen bonds are omitted for clarity. *Cg*1, *Cg*2 and *Cg*3 are the centroids of the C1–C6, N2/C8–C11/C16 and C11–C16 rings, respectively. [Symmetry codes: (v) *x*, *y* + 1, *z*; (vi) −*x* + 1, −*y* + 1, −*z*; (vii) *x*, *y* − 1, *z*.]

In the crystal of (II), the acid–base units are linked *via* C—H⋯O hydrogen bonds (C3—H3⋯O4^i^ and C4—H4⋯O3^ii^; symmetry codes as given in Table 3 ▸), forming a tape structure propagating along the *a*-axis direction (Fig. 4 ▸). The tapes are further linked into a three-dimensional network through C—H⋯O and C—H⋯Cl hydrogen bonds (C17—H17*A*⋯O2^iii^ and C17—H17*C*⋯Cl1^iv^; Table 3 ▸). In addition, π–π inter­actions are observed between the acid and base aromatic rings and between the base ring systems; the centroid–centroid distances are 3.8339 (16), 3.5056 (15) and 3.8381 (15) Å, respectively, for *Cg*1⋯*Cg*3^v^, *Cg*2⋯*Cg*2^vi^ and *Cg*2⋯*Cg*3^vi^ [symmetry codes: (v) *x*, *y* + 1, *z*; (vi) −*x* + 1, −*y* + 1, −*z*]. The acid–base units are linked *via* these π–π inter­actions, forming a ribbon structure along the *b-*axis direction (Fig. 5 ▸).

**Figure 6:**
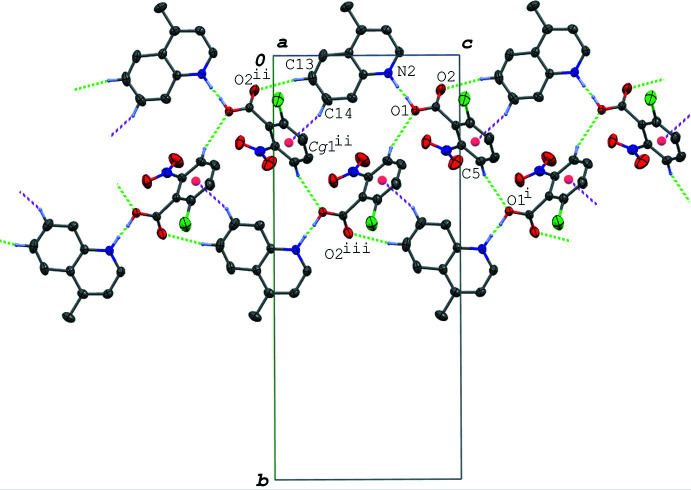
A partial packing diagram of (III) viewed along the *a* axis, showing the ribbon structure formed by the O—H⋯N/O⋯H—N and C—H⋯O hydrogen bonds (green dashed lines), and C—H⋯π inter­actions (magenta dashed lines). H atoms not involved in the inter­molecular inter­actions and the disordered O atoms of the minor component of the nitro group are omitted for clarity. [Symmetry codes: (i) *x*, −*y* + 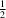 , *z* + 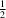 ; (ii) *x*, *y*, *z* − 1; (iii) *x*, −*y* + 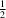 , *z* − 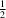 .]

**Figure 7:**
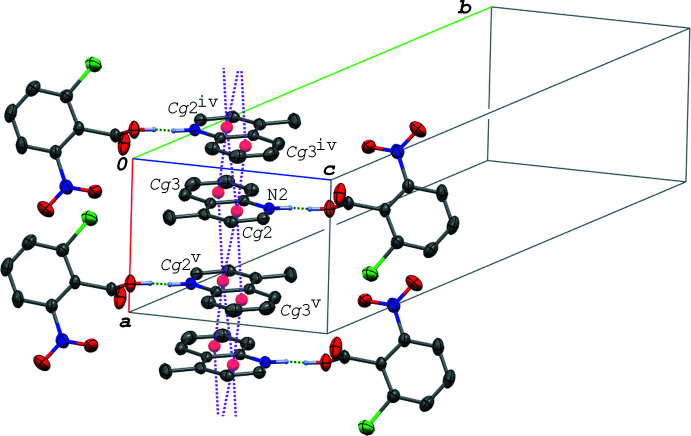
A packing diagram of (III), showing the column structure of the base mol­ecules formed *via* the π–π inter­actions (magenta dashed lines). H atoms not involved in the O—H⋯N/O⋯H—N hydrogen bonds (green dashed lines) and the disordered O atoms of the minor component of the nitro group are omitted for clarity. *Cg*2 and *Cg*3 are the centroids of the N2/C8–C11/C16 and C11–C16 rings, respectively. [Symmetry codes: (iv) −*x*, −*y*, −*z* + 1; (v) −*x* + 1, −*y*, −*z* + 1.]

In the crystal of (III), the acid–base units are linked by C—H⋯O hydrogen bonds and a C—H⋯π inter­action (C5—H5⋯O1^i^, C13—H13⋯O2^ii^ and C14—H14⋯*Cg*1^ii^; symmetry codes as in Table 4 ▸), forming a ribbon structure along the *c-*axis direction (Fig. 6 ▸). The base mol­ecules are further stacked in a column along the *a* axis *via* π–π inter­actions between the quinoline ring systems (Fig. 7 ▸), and thus the hydrogen-bonded acid–base units form a three-dimensional network. The centroid–centroid distances are 3.4323 (7), 3.4850 (7), 3.6810 (7) and 3.5878 (7) Å, respectively, for *Cg*2⋯*Cg*2^iv^, *Cg*2⋯*Cg*2^v^, *Cg*2⋯*Cg*3^iv^ and *Cg*2⋯*Cg*3^v^ [symmetry codes: (iv) −*x*, −*y*, −*z* + 1; (v) −*x* + 1, −*y*, −*z* + 1].

**Figure 8:**
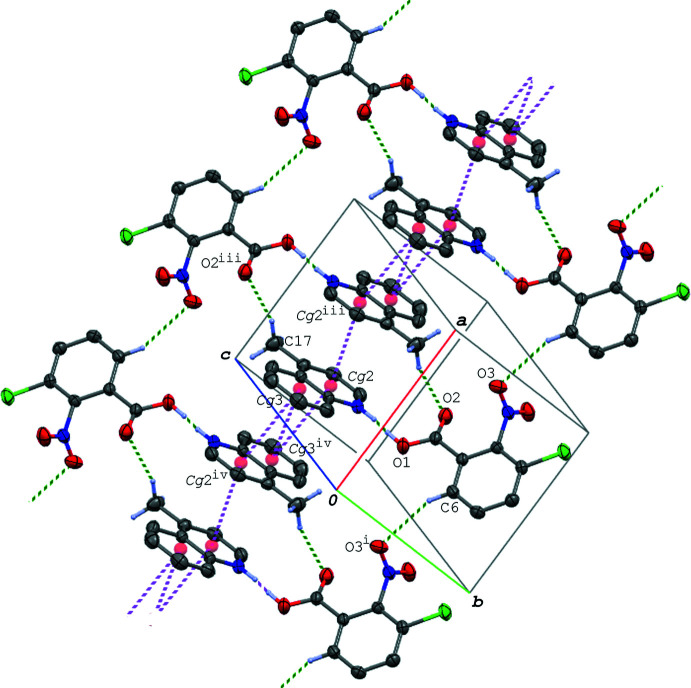
A packing diagram of (IV), showing the ribbon structure formed *via* the π–π inter­actions (magenta dashed lines), and the O—H⋯N/O⋯H—N and C—H⋯O hydrogen bonds (green dashed lines). Except for the methyl group, H atoms not involved in the hydrogen bonds are omitted for clarity. *Cg*2 and *Cg*3 are the centroids of the N2/C8–C11/C16 and C11–C16 rings, respectively. [Symmetry codes: (i) *x* − 1, *y*, *z*; (iii) −*x* + 1, −*y*, −*z* + 1; (iv) −*x*, −*y*, −*z* + 1.]

**Figure 9:**
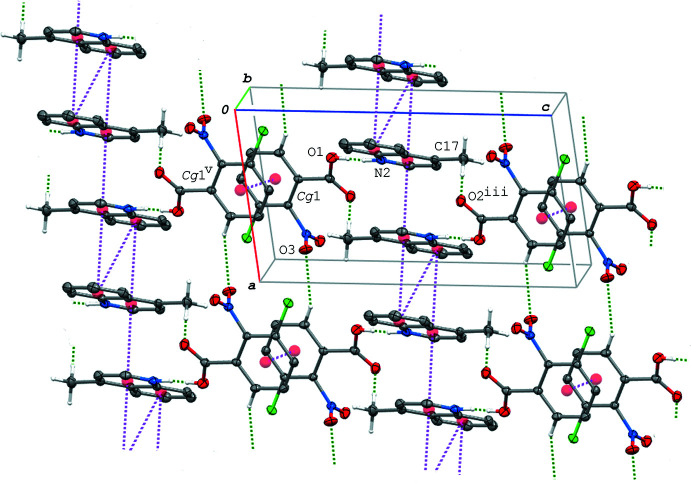
A packing diagram of (IV), showing the layer structure formed *via* the π–π inter­actions (magenta dashed lines), and the O—H⋯N/O⋯H—N and C—H⋯O hydrogen bonds (green dashed lines). Except for the methyl group, H atoms not involved in the hydrogen bonds are omitted for clarity. *Cg*1 is the centroid of the C1–C6 ring. [Symmetry codes: (iii) −*x* + 1, −*y*, −*z* + 1; (v) −*x* + 1, −*y* + 1, −*z* + 1.]

In the crystal of (IV), the hydrogen-bonded acid–base units are linked into a ribbon structure along the *a-*axis direction (Fig. 8 ▸) *via* C—H⋯O hydrogen bonds (C6—H3⋯O3^i^ and C17—H17*C*⋯O2^iii^; symmetry codes as in Table 5 ▸) and π–π inter­actions between the quinoline ring systems. The centroid–centroid distances are 3.5037 (8), 3.6022 (8) and 3.9227 (9) Å, respectively, for *Cg*2⋯*Cg*2^iii^, *Cg*2⋯*Cg*3^iv^ and *Cg*3⋯*Cg*3^iv^ [symmetry codes: (iii) −*x* + 1, −*y*, *z* + 1; (iv) −*x*, −*y*, −*z* + 1]. The ribbons are further linked into a layer parallel to the (011) plane (Fig. 9 ▸) *via* a π–π inter­action between the acid rings with a centroid–centroid distance (*Cg*1⋯*Cg*1^v^) of 3.6685 (8) Å [symmetry code: (v) −*x* + 1, −*y* + 1, −*z*]. The layers are linked by a C—H⋯O hydrogen bond (C9—H9⋯O2^ii^; Table 5 ▸).

**Figure 10:**
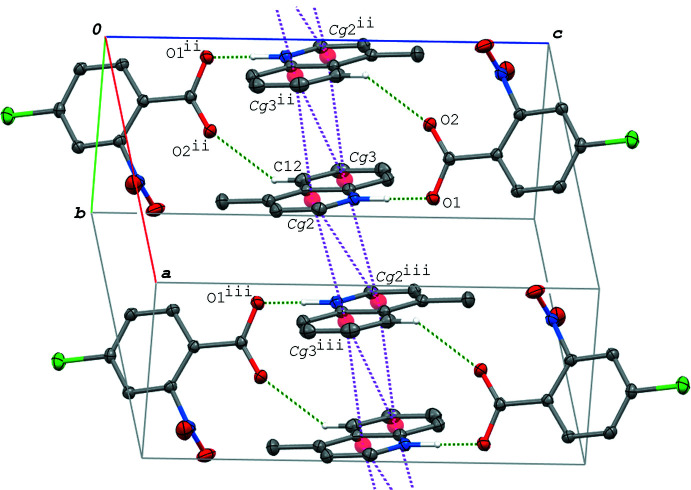
A packing diagram of (V), showing the ribbon structure formed *via* the π–π inter­actions (magenta dashed lines), and the N—H⋯O and C—H⋯O hydrogen bonds (green dashed lines). H atoms not involved in the hydrogen bonds are omitted for clarity. *Cg*2 and *Cg*3 are the centroids of the N2/C8–C11/C16 and C11–C16 rings, respectively. [Symmetry codes: (ii) −*x*, −*y* + 1, −*z* + 1; (iii) −*x* + 1, −*y* + 1, −*z* + 1.]

In the crystal of (V), the acid and base mol­ecules are arranged in a similar manner to those in (IV) as shown in Figs. 8 ▸ and 9 ▸. The hydrogen-bonded acid–base units in (V) are linked into a ribbon structure along the *a-*axis direction (Fig. 10 ▸) *via* a C—H⋯O hydrogen bond (C12—H12⋯O2^ii^; symmetry code as in Table 6 ▸) and π–π inter­actions between the quinoline ring systems. The ribbons are further linked into a layer parallel to the (011) plane *via* a π–π inter­action between the acid rings. The centroid–centroid distances of the π–π inter­actions are 3.5702 (7), 3.7751 (8), 3.7870 (8), 3.9304 (9) and 3.7719 (8) Å, respectively, for *Cg*1⋯*Cg*1^vi^, *Cg*2⋯*Cg*2^iii^, *Cg*2⋯*Cg*3^ii^, *Cg*2⋯*Cg*3^iii^ and *Cg*3⋯*Cg*3^ii^ [symmetry codes: (ii) −*x*, −*y* + 1, −*z* + 1; (iii) −*x* + 1, −*y* + 1, −*z* + 1; (iv) −*x* + 1, −*y*, −*z* + 2]. Between the layers, a C—H⋯O hydrogen bond is observed (C8—H8⋯O2^i^; Table 6 ▸).

**Figure 11:**
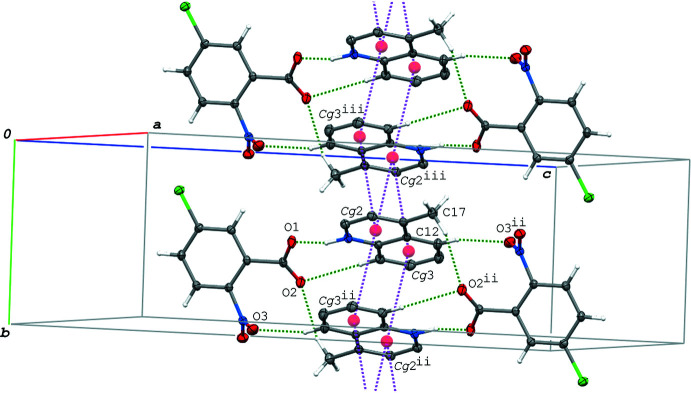
A packing diagram of (VI), showing the ribbon structure formed *via* the π–π inter­actions (magenta dashed lines), and the N—H⋯O and C—H⋯O hydrogen bonds (green dashed lines). H atoms not involved in the hydrogen bonds are omitted for clarity. *Cg*2 and *Cg*3 are the centroids of the N2/C8–C11/C16 and C11–C16 rings, respectively. [Symmetry codes: (ii) −*x* + 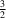 , −*y* + 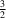 , −*z* + 1; (iii) −*x* + 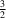 , −*y* + 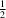 , −*z* + 1.]

Although the crystal system of (VI) (monoclinic, *C*2/*c*) is different from those of (IV) and (V) (triclinic, *P* 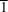 ), the mol­ecules in the crystal of (VI) are arranged in a similar manner to those in (IV) and (V). The acid–base units, which are related by an inversion centre to each other, are linked together *via* π–π inter­actions between the quinoline ring systems and C—H⋯O hydrogen bonds [*Cg*2⋯*Cg*3^ii^ = 3.8048 (7) Å; C12—H12⋯O3^ii^ and C17—H17*A*⋯O2^ii^; symmetry code as given in Table 7 ▸], forming a centrosymmetric dimeric unit. The dimeric units are further linked into a ribbon structure along the *b-*axis direction (Fig. 11 ▸) *via* other π–π inter­actions between the quinoline ring systems with *Cg*2⋯*Cg*2^iii^ = 3.4710 (6) Å and *Cg*2⋯*Cg*3^iii^ = 3.8841 (7) Å [symmetry code: (iii) −*x* + 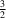 , −*y* + 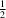 , −*z* + 1]. The ribbons are connected into a layer parallel to (10 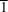 ) *via* a weak π–π inter­action between adjacent acid rings with *Cg*1⋯*Cg*1^iv^ = 3.8602 (6) Å [symmetry code: (iv) −*x* + 1, *y*, −*z* + 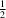 ]. Between the layers, a C—H⋯O hydrogen bond (C9—H9⋯O2^i^; Table 7 ▸) is observed.

**Figure 12:**
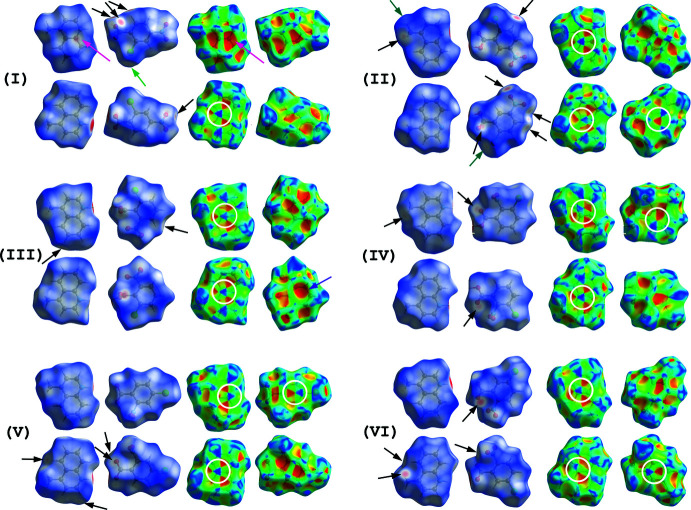
Hirshfeld surfaces [front (top) and back (bottom) views] for compounds (I)–(VI) mapped over *d*_norm_ and shape index. Each surface is viewed approximately perpendicular to the mol­ecular plane. The π–π inter­actions are shown by white circles, and the Cl⋯Cl contacts, the C—H⋯O, C—H⋯Cl, N—O⋯π and C—H⋯π inter­actions are indicated by green, black, green cyan, magenta and violet arrows, respectively.

Hirshfeld surfaces for compounds (I)–(VI) mapped over *d*_norm_ and shape index (Turner *et al.*, 2017 ▸; McKinnon *et al.*, 2004 ▸, 2007 ▸) are shown in Fig. 12 ▸. The π–π inter­actions are indicated by blue and red triangles on the shape-index surfaces (white circles in Fig. 12 ▸). On all the surfaces of the quinoline ring systems except one of the back view of (II), the π–π inter­actions between the quinoline ring systems are observed. On the surfaces of both acid and base mol­ecules of the back view of (II), the π–π inter­actions between the acid ring and the quinoline ring system are shown, while the inter­actions between the acid rings are observed on the acid ring surfaces of (IV)–(VI). The C—H⋯O inter­actions in (I)–(VI) are indicated by faint-red spots on the *d*_norm_ surfaces (black arrows). In addition, the short Cl⋯Cl contact and the N—O⋯π inter­action in (I), and the C—H⋯Cl inter­action in (II) are shown as faint-red spots on the *d*_norm_ surfaces (green, magenta and cyan arrows, respectively). On the shape-index surfaces of (I) and (III), large red areas corresponding to the N—O⋯π and C—H⋯π inter­actions (magenta and violet arrows, respectively) are observed.

## Database survey

A search of the Cambridge Structural Database (CSD Version 5.42, last update September 2021; Groom *et al.*, 2016 ▸) for organic co-crystals/salts of 4-methyl­quinoline with carb­oxy­lic acid derivatives showed one structure, namely, 4-methyl­quinoline hydrogensquarate (CSD refcode GUKWAN; Kotov *et al.*, 2018 ▸). A search for organic co-crystals/salts of 2-chloro-4-nitro­benzoic acid, 2-chloro-5-nitro­benzoic acid, 2-chloro-6-nitro­benzoic acid, 3-chloro-2-nitro­benzoic acid, 4-chloro-2-nitro­benzoic acid and 5-chloro-2-nitro­benzoic acid gave 76, 19, 0, 11, 15 and 11 structures, respectively. Limiting the search for quinoline derivatives of these compounds gave 4, 3, 0, 5, 3 and 2 compounds, namely, for 2-chloro-4-nitro­benzoic acid: 2-chloro-4-nitro­benzoic acid–6-methyl­quinoline (BUZNIW; Gotoh & Ishida, 2020 ▸), 2-chloro-4-nitro­benzoic acid–5-nitro­quinoline (NUBHEA; Gotoh & Ishida, 2019*b* ▸), 8-hy­droxy­quinolinium 2-chloro-4-nitro­benzoate (WOPDEM; Babu & Chandrasekaran, 2014 ▸), 2-chloro-4-nitro­benzoic acid–quinoline (YAGFAP; Gotoh & Ishida, 2011 ▸), for 2-chloro-5-nitro­benzoic acid: 2-chloro-5-nitro­benzoic acid–6-methyl­quinoline (BUZNOC; Gotoh & Ishida, 2020 ▸), 2-chloro-5-nitro­benzoic acid–quinoline (AJIWIA; Gotoh & Ishida, 2009 ▸), 8-hy­droxy-2-methyl­quinolinium 2-chloro-5-nitro­benzoate dihydrate (HIHPIY; Tan, 2007 ▸), for 3-chloro-2-nitro­benzoic acid: 3-chloro-2-nitro­benzoic acid–6-methyl­quinoline (BUZNUI; Gotoh & Ishida, 2020 ▸), 3-chloro-2-nitro­benzoic acid–5-nitro­quinoline (XOWVUD; Gotoh & Ishida, 2019*a* ▸), 3-chloro-2-nitro­benzoic acid–6-nitro­quinoline (XOWWAK, Gotoh & Ishida, 2019*a* ▸), 8-hy­droxy­quinolin-1-ium 3-chloro-2-nitro­benzoate (XOWWEO; Gotoh & Ishida, 2019*a* ▸), 3-chloro-2-nitro­benzoic acid–quinoline (AJIWOG, Gotoh & Ishida, 2009 ▸), for 4-chloro-2-nitro­benzoic acid: 4-chloro-2-nitro­benzoic acid–6-methyl­quinoline (BUZPAQ; Gotoh & Ishida, 2020 ▸), 4-hy­droxy­quinolin-1-ium 4-chloro-2-nitro­benzoate (WOVZOZ; Gotoh & Ishida, 2019*c* ▸), 4-chloro-2-nitro­benzoic acid–quinoline (AJIWUM; Gotoh & Ishida, 2009 ▸), and for 5-chloro-2-nitro­benzoic acid: 5-chloro-2-nitro­benzic acid–quinoline (AJIXAT, Gotoh & Ishida, 2009 ▸) and 5-chloro-2-nitro­benzoic acid–5-nitro­quinoline (NUBHIE; Gotoh & Ishida, 2019*b* ▸).

Of these compounds, AJIWOG, AJIWUM, AJIXAT, BUZNIW, BUZNUI and BUZPAQ show disordered O—H⋯N/O⋯H—N hydrogen bonds, while WOVZOZ shows a disorder structure in the O—H⋯O hydrogen bond accompanied by a keto-enol tautomerization in the base mol­ecule.

## Synthesis and crystallization

Single crystals of the title compounds (I)–(VI) were obtained by slow evaporation from aceto­nitrile solutions of 4-methyl­quinoline with the appropriate chloro-nitro­benzoic acid in a 1:1 molar ratio at room temperature [120 ml of an aceto­nitrile solution of 4-methyl­quinoline (0.20 g) and chloro-nitro­benzoic acid (0.28 g for each acid)].

## Refinement

Crystal data, data collection and structure refinement details are summarized in Table 8 ▸. All H atoms in compounds (I)–(VI) were found in difference-Fourier maps. The O-bound H atom in (II) and the N-bound H atoms in (I), (V) and (VI) were refined freely; the refined O—H and N—H distances are given in Tables 2 ▸, 3 ▸, 6 ▸ and 7 ▸. For (III) and (IV), H atoms in the N⋯H⋯O hydrogen bonds were found to be disordered over two positions in difference-Fourier maps. The positional parameters and occupancy factors were refined, with bond-length restraints of N—H = 0.88 (1) Å and O—H = 0.84 (1) Å, and with *U*_iso_(H) = 1.5*U*_eq_(N or O); the refined distances are given in Tables 4 ▸ and 5 ▸. Other H atoms were positioned geometrically (C—H = 0.95 or 0.98 Å) and treated as riding, with *U*_iso_(H) = 1.2 or 1.5*U*_eq_(C).

## Supplementary Material

Crystal structure: contains datablock(s) global, I, II, III, IV, V, VI. DOI: 10.1107/S2056989021010896/hb7991sup1.cif

Structure factors: contains datablock(s) I. DOI: 10.1107/S2056989021010896/hb7991Isup2.hkl

Supporting information file. DOI: 10.1107/S2056989021010896/hb7991Isup8.cml

Structure factors: contains datablock(s) II. DOI: 10.1107/S2056989021010896/hb7991IIsup3.hkl

Supporting information file. DOI: 10.1107/S2056989021010896/hb7991IIsup9.cml

Supporting information file. DOI: 10.1107/S2056989021010896/hb7991IIIsup10.cml

Structure factors: contains datablock(s) III. DOI: 10.1107/S2056989021010896/hb7991IIIsup4.hkl

Supporting information file. DOI: 10.1107/S2056989021010896/hb7991IVsup11.cml

Structure factors: contains datablock(s) IV. DOI: 10.1107/S2056989021010896/hb7991IVsup5.hkl

Supporting information file. DOI: 10.1107/S2056989021010896/hb7991Vsup12.cml

Structure factors: contains datablock(s) V. DOI: 10.1107/S2056989021010896/hb7991Vsup6.hkl

Supporting information file. DOI: 10.1107/S2056989021010896/hb7991VIsup13.cml

Structure factors: contains datablock(s) VI. DOI: 10.1107/S2056989021010896/hb7991VIsup7.hkl

CCDC references: 2116680, 2116679, 2116678, 2116677, 2116676, 2116675

Additional supporting information:  crystallographic information; 3D view; checkCIF report

## Figures and Tables

**Table 1:** Dihedral angles in the acid-base unit (°), hydrogen position and Δp*K*_a_ *A*, *B*, *C*, *D* and *E* are the dihedral angles between the C1–C6 ring and the N2/C8–C16 ring system, between the O1/C7/O2 plane and the N2/C8–C16 ring system, between the C1–C6 ring and the O1/C7/O2 plane, between the C1–C6 ring and the O3/N1/O4 plane, and between the N2/C8–C16 ring system and the nitro group attached to it, respectively.

	*A*	*B*	*C*	*D*	*E*	H-atom site	Δp*K*_a_
2-Chloro-4-nitro­benzoic acid							
(I)	69.15 (5)	26.60 (16)	51.29 (17)	17.77 (14)		N	3.62
*a*	3.15 (7)	43.0 (2)	39.9 (2)	12.2 (2)		O	2.86
*b*	1.11 (4)	28.59 (12)	29.36 (12)	8.24 (11)		O/N	3.16
*c*	3.94 (17)	7.5 (5)	4.3 (5)	2.5 (5)	36.2 (5)	O	0.76
2-Chloro-5-nitro­benzoic acid							
(II)	13.81 (10)	14.1 (3)	24.6 (3)	9.7 (3)		O	3.44
*a*	1.92 (4)	22.48 (14)	21.02 (14)	0.50 (13)		O	2.68
*b*	2.15 (4)	24.51 (15)	22.63 (15)	0.77 (14)		O	2.98
2-Chloro-6-nitro­benzoic acid							
(III)	61.05 (5)	35.42 (16)	84.53 (16)	21.7 (8), 14.7 (14)		O/N	4.04
3-Chloro-2-nitro­benzoic acid							
(IV)	59.45 (4)	37.30 (13)	22.39 (13)	75.20 (13)		O/N	3.84
*a*	4.71 (5)	6.18 (16)	9.22 (16)	84.97 (13)		O/N	3.08
*b*	14.50 (5)	12.55 (18)	3.14 (18)	85.04 (11)		O/N	3.38
*c*	2.59 (4)	9.95 (12)	9.45 (12)	86.14 (13)	31.67 (11)	O	0.98
*d*	10.99 (4)	12.08 (13)	2.40 (13)	88.54 (13)	5.58 (12)	O	1.42
4-Chloro-2-nitro­benzoic acid							
(V)	61.21 (5)	67.42 (14)	10.22 (14)	80.76 (15)		N	3.69
*a*	31.65 (4)	18.77 (13)	13.71 (13)	76.44 (17)		O/N	2.93
*b*	30.39 (9)	21.7 (3)	16.4 (3)	74.4 (3)		O/N	3.23
5-Chloro-2-nitro­benzoic acid							
(VI)	58.90 (4)	23.54 (13)	35.43 (13)	57.13 (11)		N	3.80
*a*	54.43 (5)	5.41 (15)	49.95 (15)	33.31 (13)		O/N	3.04
*c*	37.37 (6)	2.9 (2)	40.3 (2)	47.12 (19)	11.3 (2)	O	0.94

Notes: *a*: quinoline compounds (Gotoh & Ishida, 2009 ▸, 2011 ▸), *b*: 6-methyl­quinoline compounds (Gotoh & Ishida, 2020 ▸), *c*: 5-nitro­quinoline compounds (Gotoh & Ishida, 2019*a* ▸,*b* ▸) and *d*: 6-nitro­quinoline–3-chloro-2-nitro­benzoic acid (Gotoh & Ishida, 2019*a* ▸).

**Table 2:** Hydrogen-bond geometry (Å, °) for (I) *Cg*3 is the centroid of the C11–C16 ring.

*D*—H⋯*A*	*D*—H	H⋯*A*	*D*⋯*A*	*D*—H⋯*A*
N2—H2⋯O1	0.900 (19)	1.678 (19)	2.5652 (14)	167.7 (18)
C6—H6⋯O2^i^	0.95	2.39	3.3066 (16)	163
C8—H8⋯O3^ii^	0.95	2.56	3.4199 (16)	151
C9—H9⋯O2^iii^	0.95	2.44	3.3360 (16)	158
C15—H15⋯O2	0.95	2.36	3.2835 (17)	163
N1—O3⋯*Cg*3^iv^	1.22 (1)	3.26 (1)	4.3171 (13)	145 (1)

Symmetry codes: (i) -x, -y+1, -z+1; (ii) -x, -y+1, -z+2; (iii) x, y+1, z; (iv) x, y-1, z+1.

**Table 3:** Hydrogen-bond geometry (Å, °) for (II)

*D*—H⋯*A*	*D*—H	H⋯*A*	*D*⋯*A*	*D*—H⋯*A*
O1—H1⋯N2	0.91 (7)	1.68 (7)	2.556 (3)	162 (7)
C3—H3⋯O4^i^	0.95	2.40	3.280 (4)	154
C4—H4⋯O3^ii^	0.95	2.54	3.188 (3)	126
C17—H17*A*⋯O2^iii^	0.98	2.57	3.479 (4)	155
C17—H17*C*⋯Cl1^iv^	0.98	2.81	3.535 (4)	131

Symmetry codes: (i) x-1, y, z; (ii) -x+1, -y+3, -z+1; (iii) x, y-1, z; (iv) -x, -y+1, -z.

**Table 4:** Hydrogen-bond geometry (Å, °) for (III) *Cg*1 is the centroid of the C1–C6 ring.

*D*—H⋯*A*	*D*—H	H⋯*A*	*D*⋯*A*	*D*—H⋯*A*
O1—H1⋯N2	0.84 (4)	1.71 (4)	2.5485 (13)	177 (6)
N2—H2⋯O1	0.89 (2)	1.66 (2)	2.5485 (13)	176 (2)
C5—H5⋯O1^i^	0.95	2.49	3.1489 (15)	126
C13—H13⋯O2^ii^	0.95	2.36	3.2889 (17)	165
C14—H14⋯*Cg*1^ii^	0.95	2.89	3.6596 (15)	138

Symmetry codes: (i) x, -y+{\script{1\over 2}}, z+{\script{1\over 2}}; (ii) x, y, z-1.

**Table 5:** Hydrogen-bond geometry (Å, °) for (IV)

*D*—H⋯*A*	*D*—H	H⋯*A*	*D*⋯*A*	*D*—H⋯*A*
O1—H1⋯N2	0.84 (3)	1.70 (3)	2.5364 (13)	175 (3)
N2—H2⋯O1	0.89 (2)	1.65 (2)	2.5364 (13)	175 (3)
C6—H6⋯O3^i^	0.95	2.59	3.4705 (14)	155
C9—H9⋯O2^ii^	0.95	2.41	3.1739 (15)	137
C17—H17*C*⋯O2^iii^	0.98	2.47	3.4155 (17)	162

Symmetry codes: (i) x-1, y, z; (ii) -x+1, -y+1, -z+1; (iii) -x+1, -y, -z+1.

**Table 6:** Hydrogen-bond geometry (Å, °) for (V)

*D*—H⋯*A*	*D*—H	H⋯*A*	*D*⋯*A*	*D*—H⋯*A*
N2—H2⋯O1	1.06 (2)	1.50 (2)	2.5568 (13)	179 (4)
C8—H8⋯O2^i^	0.95	2.56	3.2779 (16)	132
C12—H12⋯O2^ii^	0.95	2.52	3.3391 (18)	144

Symmetry codes: (i) -x+1, -y, -z+1; (ii) -x, -y+1, -z+1.

**Table 7:** Hydrogen-bond geometry (Å, °) for (VI)

*D*—H⋯*A*	*D*—H	H⋯*A*	*D*⋯*A*	*D*—H⋯*A*
N2—H2⋯O1	1.03 (2)	1.52 (2)	2.5252 (11)	165 (2)
C9—H9⋯O2^i^	0.95	2.34	3.2856 (13)	171
C12—H12⋯O3^ii^	0.95	2.58	3.5065 (14)	166
C15—H15⋯O2	0.95	2.57	3.4583 (13)	155
C17—H17*A*⋯O2^ii^	0.98	2.41	3.3524 (16)	160

Symmetry codes: (i) x+{\script{1\over 2}}, y-{\script{1\over 2}}, z; (ii) -x+{\script{3\over 2}}, -y+{\script{3\over 2}}, -z+1.

**Table:** 

	(I)	(II)	(III)
Crystal data			
Chemical formula	C_10_H_10_N^+^·C_7_H_3_ClNO_4_^−^	C_10_H_9_N·C_7_H_4_ClNO_4_	C_10_H_9.63_N^0.63+^·C_7_H_3.37_ClNO_4_^0.63−^
*M*_r_	344.75	344.75	344.75
Crystal system, space group	Triclinic, *P*\overline{1}	Triclinic, *P*\overline{1}	Monoclinic, *P*2_1_/*c*
Temperature (K)	185	185	185
*a*, *b*, *c* (Å)	8.6975 (4), 9.2527 (4), 10.1865 (5)	7.6353 (4), 9.3827 (6), 11.3756 (7)	6.6401 (3), 23.2126 (5), 10.3386 (3)
α, β, γ (°)	72.7483 (15), 86.4281 (16), 74.5728 (15)	91.453 (3), 95.204 (3), 107.773 (3)	90, 99.3926 (15), 90
*V* (Å^3^)	754.55 (6)	771.65 (8)	1572.16 (9)
*Z*	2	2	4
Radiation type	Mo *K*α	Mo *K*α	Mo *K*α
μ (mm^−1^)	0.28	0.27	0.27
Crystal size (mm)	0.55 × 0.50 × 0.32	0.30 × 0.25 × 0.05	0.35 × 0.28 × 0.25

Data collection			
Diffractometer	Rigaku R-AXIS RAPIDII	Rigaku R-AXIS RAPIDII	Rigaku R-AXIS RAPIDII
Absorption correction	Numerical (*NUMABS*; Higashi, 1999 ▸)	Numerical (*NUMABS*; Higashi, 1999 ▸)	Numerical (*NUMABS*; Higashi, 1999 ▸)
*T*_min_, *T*_max_	0.868, 0.915	0.938, 0.986	0.909, 0.935
No. of measured, independent and observed [*I* > 2σ(*I*)] reflections	22243, 4404, 3822	14544, 4486, 2563	32362, 4588, 3854
*R*_int_	0.043	0.038	0.022
(sin θ/λ)_max_ (Å^−1^)	0.704	0.703	0.704

Refinement			
*R*[*F* ^2^ > 2σ(*F* ^2^)], *wR*(*F* ^2^), *S*	0.042, 0.122, 1.13	0.068, 0.257, 1.19	0.044, 0.125, 1.07
No. of reflections	4404	4486	4588
No. of parameters	222	222	244
No. of restraints	0	0	2
H-atom treatment	H atoms treated by a mixture of independent and constrained refinement	H atoms treated by a mixture of independent and constrained refinement	H atoms treated by a mixture of independent and constrained refinement
Δρ_max_, Δρ_min_ (e Å^−3^)	0.44, −0.28	0.91, −0.58	0.52, −0.40

**Table:** 

	(IV)	(V)	(VI)
Crystal data			
Chemical formula	C_10_H_9.54_N^0.54+^·C_7_H_3.46_ClNO_4_^0.54−^	C_10_H_10_N^+^·C_7_H_3_ClNO_4_^−^	C_10_H_10_N^+^.C_7_H_3_ClNO_4_^−^
*M*_r_	344.75	344.75	344.75
Crystal system, space group	Triclinic, *P*\overline{1}	Triclinic, *P*\overline{1}	Monoclinic, *C*2/*c*
Temperature (K)	185	185	190
*a*, *b*, *c* (Å)	7.5234 (10), 7.8017 (11), 13.6341 (17)	7.6858 (3), 8.3615 (3), 13.5746 (5)	16.2625 (10), 7.5099 (4), 25.3105 (15)
α, β, γ (°)	80.934 (4), 80.227 (3), 89.150 (4)	82.5485 (13), 80.8927 (12), 65.0929 (11)	90, 99.4086 (19), 90
*V* (Å^3^)	778.73 (18)	779.33 (5)	3049.6 (3)
*Z*	2	2	8
Radiation type	Mo *K*α	Mo *K*α	Mo *K*α
μ (mm^−1^)	0.27	0.27	0.28
Crystal size (mm)	0.35 × 0.29 × 0.22	0.51 × 0.45 × 0.15	0.30 × 0.21 × 0.12

Data collection			
Diffractometer	Rigaku R-AXIS RAPIDII	Rigaku R-AXIS RAPIDII	Rigaku R-AXIS RAPIDII
Absorption correction	Numerical (*NUMABS*; Higashi, 1999 ▸)	Numerical (*NUMABS*; Higashi, 1999 ▸)	Numerical (*NUMABS*; Higashi, 1999 ▸)
*T*_min_, *T*_max_	0.914, 0.942	0.868, 0.960	0.916, 0.968
No. of measured, independent and observed [*I* > 2σ(*I*)] reflections	16767, 4544, 4017	18635, 3566, 3290	29037, 4457, 3913
*R*_int_	0.028	0.027	0.022
(sin θ/λ)_max_ (Å^−1^)	0.704	0.649	0.703

Refinement			
*R*[*F* ^2^ > 2σ(*F* ^2^)], *wR*(*F* ^2^), *S*	0.036, 0.103, 1.07	0.036, 0.102, 1.04	0.036, 0.099, 1.05
No. of reflections	4544	3566	4457
No. of parameters	225	222	222
No. of restraints	2	0	0
H-atom treatment	H atoms treated by a mixture of independent and constrained refinement	H atoms treated by a mixture of independent and constrained refinement	H atoms treated by a mixture of independent and constrained refinement
Δρ_max_, Δρ_min_ (e Å^−3^)	0.44, −0.38	0.38, −0.18	0.47, −0.16

Computer programs: *PROCESS-AUTO* (Rigaku, 2006 ▸), *CrystalStructure* (Rigaku, 2018 ▸), *SHELXS97* (Sheldrick, 2008 ▸), *SIR92* (Altomare *et al.*, 1993 ▸), *SHELXL2018/3* (Sheldrick, 2015 ▸), *ORTEP-3 for Windows* (Farrugia, 2012 ▸), *Mercury* (Macrae *et al.*, 2020 ▸) and *PLATON* (Spek, 2020 ▸).

## supplementary crystallographic information

### 4-Methylquinolinium 2-chloro-4-nitrobenzoate (I) Crystal data

**Table:**

C_10_H_10_N^+^·C_7_H_3_ClNO_4_^−^	*Z* = 2
*M**_r_* = 344.75	*F*(000) = 356.00
Triclinic, *P*1	*D*_x_ = 1.517 Mg m^−^^3^
*a* = 8.6975 (4) Å	Mo *K*α radiation, λ = 0.71075 Å
*b* = 9.2527 (4) Å	Cell parameters from 20962 reflections
*c* = 10.1865 (5) Å	θ = 3.2–30.2°
α = 72.7483 (15)°	µ = 0.28 mm^−^^1^
β = 86.4281 (16)°	*T* = 185 K
γ = 74.5728 (15)°	Block, colorless
*V* = 754.55 (6) Å^3^	0.55 × 0.50 × 0.32 mm

### 4-Methylquinolinium 2-chloro-4-nitrobenzoate (I) Data collection

**Table:**

Rigaku R-AXIS RAPIDII diffractometer	3822 reflections with *I* > 2σ(*I*)
Detector resolution: 10.000 pixels mm^-1^	*R*_int_ = 0.043
ω scans	θ_max_ = 30.0°, θ_min_ = 3.2°
Absorption correction: numerical (NUMABS; Higashi, 1999)	*h* = −12→12
*T*_min_ = 0.868, *T*_max_ = 0.915	*k* = −13→13
22243 measured reflections	*l* = −14→14
4404 independent reflections	

### 4-Methylquinolinium 2-chloro-4-nitrobenzoate (I) Refinement

**Table:**

Refinement on *F*^2^	Secondary atom site location: difference Fourier map
*R*[*F*^2^ > 2σ(*F*^2^)] = 0.042	Hydrogen site location: mixed
*wR*(*F*^2^) = 0.122	H atoms treated by a mixture of independent and constrained refinement
*S* = 1.13	*w* = 1/[σ^2^(*F*_o_^2^) + (0.0733*P*)^2^ + 0.0819*P*] where *P* = (*F*_o_^2^ + 2*F*_c_^2^)/3
4404 reflections	(Δ/σ)_max_ < 0.001
222 parameters	Δρ_max_ = 0.44 e Å^−^^3^
0 restraints	Δρ_min_ = −0.28 e Å^−^^3^
Primary atom site location: structure-invariant direct methods	

### 4-Methylquinolinium 2-chloro-4-nitrobenzoate (I) Special details

**Table:**

Geometry. All esds (except the esd in the dihedral angle between two l.s. planes) are estimated using the full covariance matrix. The cell esds are taken into account individually in the estimation of esds in distances, angles and torsion angles; correlations between esds in cell parameters are only used when they are defined by crystal symmetry. An approximate (isotropic) treatment of cell esds is used for estimating esds involving l.s. planes.

### 4-Methylquinolinium 2-chloro-4-nitrobenzoate (I) Fractional atomic coordinates and isotropic or equivalent isotropic displacement parameters (Å^2^)

**Table:**

	*x*	*y*	*z*	*U*_iso_*/*U*_eq_	
Cl1	0.32990 (4)	0.49410 (3)	0.93689 (3)	0.03079 (11)	
O1	0.14650 (13)	0.72397 (10)	0.67810 (9)	0.0348 (2)	
O2	0.17832 (14)	0.56790 (11)	0.54222 (9)	0.0379 (2)	
O3	0.06312 (12)	0.05267 (11)	1.18988 (10)	0.0363 (2)	
O4	−0.15473 (12)	0.09130 (11)	1.07952 (11)	0.0371 (2)	
N1	−0.02872 (13)	0.12105 (11)	1.09235 (11)	0.0273 (2)	
N2	0.22641 (12)	0.95808 (11)	0.50904 (10)	0.0252 (2)	
H2	0.193 (2)	0.874 (2)	0.558 (2)	0.058 (6)*	
C1	0.10772 (13)	0.47157 (12)	0.77222 (11)	0.0220 (2)	
C2	0.17864 (13)	0.42010 (12)	0.90230 (11)	0.0222 (2)	
C3	0.13592 (13)	0.30429 (12)	1.00803 (11)	0.0239 (2)	
H3	0.187120	0.267833	1.095810	0.029*	
C4	0.01662 (13)	0.24410 (12)	0.98114 (12)	0.0239 (2)	
C5	−0.05810 (14)	0.29108 (13)	0.85435 (12)	0.0259 (2)	
H5	−0.140805	0.247959	0.839335	0.031*	
C6	−0.00900 (14)	0.40299 (13)	0.74954 (12)	0.0251 (2)	
H6	−0.055755	0.433693	0.660379	0.030*	
C7	0.14979 (14)	0.59753 (13)	0.65289 (12)	0.0241 (2)	
C8	0.18122 (15)	1.08320 (14)	0.55384 (12)	0.0274 (2)	
H8	0.108530	1.083030	0.627431	0.033*	
C9	0.23804 (14)	1.21539 (13)	0.49531 (12)	0.0268 (2)	
H9	0.203126	1.304456	0.528283	0.032*	
C10	0.34486 (14)	1.21633 (13)	0.38964 (11)	0.0240 (2)	
C11	0.39172 (13)	1.08266 (13)	0.33915 (11)	0.0239 (2)	
C12	0.49945 (15)	1.07123 (15)	0.22966 (13)	0.0300 (2)	
H12	0.544342	1.156119	0.185742	0.036*	
C13	0.53934 (17)	0.93898 (17)	0.18668 (14)	0.0360 (3)	
H13	0.611735	0.933167	0.113483	0.043*	
C14	0.47410 (17)	0.81181 (17)	0.24992 (15)	0.0361 (3)	
H14	0.501707	0.721573	0.218226	0.043*	
C15	0.37123 (16)	0.81710 (14)	0.35670 (13)	0.0300 (2)	
H15	0.328586	0.730472	0.399948	0.036*	
C16	0.32896 (13)	0.95269 (13)	0.40207 (11)	0.0240 (2)	
C17	0.41066 (16)	1.35602 (14)	0.32995 (13)	0.0297 (2)	
H17A	0.527472	1.321940	0.331657	0.045*	
H17B	0.375456	1.429869	0.384385	0.045*	
H17C	0.372032	1.407486	0.234819	0.045*	

### 4-Methylquinolinium 2-chloro-4-nitrobenzoate (I) Atomic displacement parameters (Å^2^)

**Table:**

	*U*^11^	*U*^22^	*U*^33^	*U*^12^	*U*^13^	*U*^23^
Cl1	0.03215 (17)	0.03700 (18)	0.02937 (16)	−0.01980 (13)	0.00018 (11)	−0.00912 (12)
O1	0.0553 (6)	0.0235 (4)	0.0299 (4)	−0.0195 (4)	0.0110 (4)	−0.0083 (3)
O2	0.0618 (7)	0.0351 (5)	0.0256 (4)	−0.0271 (5)	0.0103 (4)	−0.0110 (4)
O3	0.0435 (5)	0.0304 (5)	0.0294 (5)	−0.0118 (4)	0.0015 (4)	0.0013 (4)
O4	0.0384 (5)	0.0358 (5)	0.0410 (5)	−0.0222 (4)	0.0093 (4)	−0.0076 (4)
N1	0.0320 (5)	0.0225 (4)	0.0283 (5)	−0.0115 (4)	0.0071 (4)	−0.0059 (4)
N2	0.0284 (5)	0.0233 (4)	0.0237 (4)	−0.0109 (4)	0.0018 (4)	−0.0031 (4)
C1	0.0248 (5)	0.0192 (4)	0.0228 (5)	−0.0082 (4)	0.0023 (4)	−0.0055 (4)
C2	0.0233 (5)	0.0219 (5)	0.0243 (5)	−0.0094 (4)	0.0020 (4)	−0.0078 (4)
C3	0.0262 (5)	0.0228 (5)	0.0224 (5)	−0.0074 (4)	0.0012 (4)	−0.0053 (4)
C4	0.0266 (5)	0.0186 (5)	0.0260 (5)	−0.0087 (4)	0.0048 (4)	−0.0040 (4)
C5	0.0264 (5)	0.0230 (5)	0.0304 (6)	−0.0117 (4)	0.0003 (4)	−0.0062 (4)
C6	0.0283 (5)	0.0229 (5)	0.0248 (5)	−0.0102 (4)	−0.0024 (4)	−0.0044 (4)
C7	0.0273 (5)	0.0223 (5)	0.0240 (5)	−0.0111 (4)	0.0012 (4)	−0.0049 (4)
C8	0.0307 (6)	0.0265 (5)	0.0246 (5)	−0.0113 (4)	0.0052 (4)	−0.0044 (4)
C9	0.0313 (6)	0.0221 (5)	0.0269 (5)	−0.0092 (4)	0.0032 (4)	−0.0056 (4)
C10	0.0246 (5)	0.0220 (5)	0.0235 (5)	−0.0080 (4)	−0.0016 (4)	−0.0016 (4)
C11	0.0240 (5)	0.0245 (5)	0.0217 (5)	−0.0085 (4)	−0.0012 (4)	−0.0024 (4)
C12	0.0294 (6)	0.0327 (6)	0.0265 (5)	−0.0109 (5)	0.0032 (4)	−0.0049 (5)
C13	0.0355 (7)	0.0431 (7)	0.0315 (6)	−0.0110 (6)	0.0085 (5)	−0.0148 (5)
C14	0.0392 (7)	0.0349 (6)	0.0389 (7)	−0.0094 (5)	0.0037 (5)	−0.0184 (5)
C15	0.0336 (6)	0.0262 (5)	0.0324 (6)	−0.0105 (5)	0.0001 (5)	−0.0093 (5)
C16	0.0254 (5)	0.0238 (5)	0.0228 (5)	−0.0083 (4)	−0.0014 (4)	−0.0046 (4)
C17	0.0326 (6)	0.0240 (5)	0.0314 (6)	−0.0128 (5)	0.0027 (5)	−0.0020 (4)

### 4-Methylquinolinium 2-chloro-4-nitrobenzoate (I) Geometric parameters (Å, º)

**Table:**

Cl1—C2	1.7331 (11)	C8—C9	1.3957 (15)
O1—C7	1.2628 (13)	C8—H8	0.9500
O2—C7	1.2329 (14)	C9—C10	1.3770 (16)
O3—N1	1.2176 (14)	C9—H9	0.9500
O4—N1	1.2221 (14)	C10—C11	1.4294 (16)
N1—C4	1.4674 (14)	C10—C17	1.5007 (15)
N2—C8	1.3236 (16)	C11—C16	1.4155 (15)
N2—C16	1.3686 (15)	C11—C12	1.4213 (17)
N2—H2	0.90 (2)	C12—C13	1.3721 (19)
C1—C2	1.3908 (15)	C12—H12	0.9500
C1—C6	1.3948 (15)	C13—C14	1.408 (2)
C1—C7	1.5169 (14)	C13—H13	0.9500
C2—C3	1.3869 (15)	C14—C15	1.3686 (19)
C3—C4	1.3766 (16)	C14—H14	0.9500
C3—H3	0.9500	C15—C16	1.4142 (16)
C4—C5	1.3787 (17)	C15—H15	0.9500
C5—C6	1.3846 (15)	C17—H17A	0.9800
C5—H5	0.9500	C17—H17B	0.9800
C6—H6	0.9500	C17—H17C	0.9800
			
O3—N1—O4	124.18 (10)	C10—C9—C8	119.81 (11)
O3—N1—C4	118.01 (10)	C10—C9—H9	120.1
O4—N1—C4	117.78 (10)	C8—C9—H9	120.1
C8—N2—C16	121.80 (10)	C9—C10—C11	118.97 (10)
C8—N2—H2	115.9 (13)	C9—C10—C17	119.91 (11)
C16—N2—H2	122.1 (13)	C11—C10—C17	121.12 (10)
C2—C1—C6	118.15 (10)	C16—C11—C12	117.55 (11)
C2—C1—C7	124.07 (10)	C16—C11—C10	118.54 (10)
C6—C1—C7	117.78 (10)	C12—C11—C10	123.91 (10)
C3—C2—C1	121.81 (10)	C13—C12—C11	120.75 (12)
C3—C2—Cl1	117.20 (9)	C13—C12—H12	119.6
C1—C2—Cl1	120.94 (8)	C11—C12—H12	119.6
C4—C3—C2	117.58 (10)	C12—C13—C14	120.68 (12)
C4—C3—H3	121.2	C12—C13—H13	119.7
C2—C3—H3	121.2	C14—C13—H13	119.7
C3—C4—C5	123.04 (10)	C15—C14—C13	120.54 (12)
C3—C4—N1	117.88 (10)	C15—C14—H14	119.7
C5—C4—N1	119.05 (10)	C13—C14—H14	119.7
C4—C5—C6	118.01 (10)	C14—C15—C16	119.41 (12)
C4—C5—H5	121.0	C14—C15—H15	120.3
C6—C5—H5	121.0	C16—C15—H15	120.3
C5—C6—C1	121.33 (11)	N2—C16—C15	119.51 (10)
C5—C6—H6	119.3	N2—C16—C11	119.42 (10)
C1—C6—H6	119.3	C15—C16—C11	121.08 (11)
O2—C7—O1	127.32 (10)	C10—C17—H17A	109.5
O2—C7—C1	117.26 (9)	C10—C17—H17B	109.5
O1—C7—C1	115.38 (10)	H17A—C17—H17B	109.5
N2—C8—C9	121.44 (11)	C10—C17—H17C	109.5
N2—C8—H8	119.3	H17A—C17—H17C	109.5
C9—C8—H8	119.3	H17B—C17—H17C	109.5
			
C6—C1—C2—C3	−0.18 (17)	C16—N2—C8—C9	−0.99 (18)
C7—C1—C2—C3	−179.94 (10)	N2—C8—C9—C10	−0.74 (19)
C6—C1—C2—Cl1	177.53 (8)	C8—C9—C10—C11	1.89 (18)
C7—C1—C2—Cl1	−2.23 (16)	C8—C9—C10—C17	−177.80 (11)
C1—C2—C3—C4	−1.77 (17)	C9—C10—C11—C16	−1.39 (16)
Cl1—C2—C3—C4	−179.56 (8)	C17—C10—C11—C16	178.29 (11)
C2—C3—C4—C5	1.57 (17)	C9—C10—C11—C12	179.23 (11)
C2—C3—C4—N1	179.74 (9)	C17—C10—C11—C12	−1.09 (18)
O3—N1—C4—C3	−17.00 (15)	C16—C11—C12—C13	0.49 (18)
O4—N1—C4—C3	164.68 (11)	C10—C11—C12—C13	179.88 (12)
O3—N1—C4—C5	161.25 (11)	C11—C12—C13—C14	0.1 (2)
O4—N1—C4—C5	−17.08 (16)	C12—C13—C14—C15	−0.9 (2)
C3—C4—C5—C6	0.61 (18)	C13—C14—C15—C16	0.9 (2)
N1—C4—C5—C6	−177.54 (10)	C8—N2—C16—C15	−178.90 (11)
C4—C5—C6—C1	−2.68 (18)	C8—N2—C16—C11	1.46 (17)
C2—C1—C6—C5	2.47 (17)	C14—C15—C16—N2	−179.84 (12)
C7—C1—C6—C5	−177.75 (10)	C14—C15—C16—C11	−0.21 (19)
C2—C1—C7—O2	130.38 (13)	C12—C11—C16—N2	179.17 (10)
C6—C1—C7—O2	−49.38 (16)	C10—C11—C16—N2	−0.25 (16)
C2—C1—C7—O1	−51.76 (16)	C12—C11—C16—C15	−0.47 (17)
C6—C1—C7—O1	128.48 (12)	C10—C11—C16—C15	−179.89 (10)

### 4-Methylquinolinium 2-chloro-4-nitrobenzoate (I) Hydrogen-bond geometry (Å, º)

*Cg*3 is the centroid of the C11–C16 ring.

**Table:**

*D*—H···*A*	*D*—H	H···*A*	*D*···*A*	*D*—H···*A*
N2—H2···O1	0.900 (19)	1.678 (19)	2.5652 (14)	167.7 (18)
C6—H6···O2^i^	0.95	2.39	3.3066 (16)	163
C8—H8···O3^ii^	0.95	2.56	3.4199 (16)	151
C9—H9···O2^iii^	0.95	2.44	3.3360 (16)	158
C15—H15···O2	0.95	2.36	3.2835 (17)	163
N1—O3···*Cg*3^iv^	1.22 (1)	3.26 (1)	4.3171 (13)	145 (1)

Symmetry codes: (i) −*x*, −*y*+1, −*z*+1; (ii) −*x*, −*y*+1, −*z*+2; (iii) *x*, *y*+1, *z*; (iv) *x*, *y*−1, *z*+1.

### 4-Methylquinoline–2-chloro-5-nitrobenzoic acid (1/1) (II) Crystal data

**Table:**

C_10_H_9_N·C_7_H_4_ClNO_4_	*Z* = 2
*M**_r_* = 344.75	*F*(000) = 356.00
Triclinic, *P*1	*D*_x_ = 1.484 Mg m^−^^3^
*a* = 7.6353 (4) Å	Mo *K*α radiation, λ = 0.71075 Å
*b* = 9.3827 (6) Å	Cell parameters from 9512 reflections
*c* = 11.3756 (7) Å	θ = 3.0–30.1°
α = 91.453 (3)°	µ = 0.27 mm^−^^1^
β = 95.204 (3)°	*T* = 185 K
γ = 107.773 (3)°	Platelet, colorless
*V* = 771.65 (8) Å^3^	0.30 × 0.25 × 0.05 mm

### 4-Methylquinoline–2-chloro-5-nitrobenzoic acid (1/1) (II) Data collection

**Table:**

Rigaku R-AXIS RAPIDII diffractometer	2563 reflections with *I* > 2σ(*I*)
Detector resolution: 10.000 pixels mm^-1^	*R*_int_ = 0.038
ω scans	θ_max_ = 30.0°, θ_min_ = 3.0°
Absorption correction: numerical (NUMABS; Higashi, 1999)	*h* = −9→10
*T*_min_ = 0.938, *T*_max_ = 0.986	*k* = −13→13
14544 measured reflections	*l* = −15→15
4486 independent reflections	

### 4-Methylquinoline–2-chloro-5-nitrobenzoic acid (1/1) (II) Refinement

**Table:**

Refinement on *F*^2^	Secondary atom site location: difference Fourier map
*R*[*F*^2^ > 2σ(*F*^2^)] = 0.068	Hydrogen site location: mixed
*wR*(*F*^2^) = 0.257	H atoms treated by a mixture of independent and constrained refinement
*S* = 1.19	*w* = 1/[σ^2^(*F*_o_^2^) + (0.1416*P*)^2^] where *P* = (*F*_o_^2^ + 2*F*_c_^2^)/3
4486 reflections	(Δ/σ)_max_ < 0.001
222 parameters	Δρ_max_ = 0.91 e Å^−^^3^
0 restraints	Δρ_min_ = −0.58 e Å^−^^3^
Primary atom site location: structure-invariant direct methods	

### 4-Methylquinoline–2-chloro-5-nitrobenzoic acid (1/1) (II) Special details

**Table:**

Geometry. All esds (except the esd in the dihedral angle between two l.s. planes) are estimated using the full covariance matrix. The cell esds are taken into account individually in the estimation of esds in distances, angles and torsion angles; correlations between esds in cell parameters are only used when they are defined by crystal symmetry. An approximate (isotropic) treatment of cell esds is used for estimating esds involving l.s. planes.

### 4-Methylquinoline–2-chloro-5-nitrobenzoic acid (1/1) (II) Fractional atomic coordinates and isotropic or equivalent isotropic displacement parameters (Å^2^)

**Table:**

	*x*	*y*	*z*	*U*_iso_*/*U*_eq_	
Cl1	−0.03754 (10)	0.97793 (9)	0.28787 (8)	0.0688 (3)	
O1	0.4426 (3)	0.8113 (2)	0.27937 (18)	0.0550 (5)	
H1	0.401 (10)	0.721 (8)	0.238 (6)	0.17 (3)*	
O2	0.1900 (4)	0.8238 (2)	0.1691 (2)	0.0705 (7)	
O3	0.7457 (3)	1.4293 (2)	0.54603 (18)	0.0510 (5)	
O4	0.8405 (2)	1.2413 (2)	0.50002 (19)	0.0515 (5)	
N1	0.7203 (3)	1.3046 (2)	0.49943 (18)	0.0386 (5)	
N2	0.3941 (3)	0.5596 (2)	0.1685 (2)	0.0465 (5)	
C1	0.3346 (4)	1.0135 (3)	0.3225 (2)	0.0384 (5)	
C2	0.1888 (3)	1.0712 (3)	0.3399 (2)	0.0420 (5)	
C3	0.2183 (4)	1.2039 (3)	0.4056 (2)	0.0445 (6)	
H3	0.117494	1.240795	0.416034	0.053*	
C4	0.3935 (3)	1.2831 (3)	0.4562 (2)	0.0384 (5)	
H4	0.415578	1.375700	0.499746	0.046*	
C5	0.5360 (3)	1.2245 (2)	0.4418 (2)	0.0346 (5)	
C6	0.5106 (3)	1.0921 (2)	0.3766 (2)	0.0366 (5)	
H6	0.611816	1.054882	0.368761	0.044*	
C7	0.3130 (4)	0.8720 (3)	0.2488 (2)	0.0462 (6)	
C8	0.2387 (4)	0.4902 (3)	0.1035 (2)	0.0493 (6)	
H8	0.146250	0.538936	0.096541	0.059*	
C9	0.1990 (4)	0.3519 (3)	0.0444 (2)	0.0467 (6)	
H9	0.083758	0.308531	−0.001836	0.056*	
C10	0.3317 (4)	0.2774 (3)	0.0539 (2)	0.0460 (6)	
C11	0.5048 (3)	0.3480 (2)	0.12444 (19)	0.0352 (5)	
C12	0.6504 (4)	0.2871 (4)	0.1402 (3)	0.0545 (7)	
H12	0.636763	0.192542	0.102077	0.065*	
C13	0.8077 (5)	0.3570 (4)	0.2068 (3)	0.0643 (9)	
H13	0.903299	0.311688	0.216432	0.077*	
C14	0.8312 (4)	0.4947 (4)	0.2615 (3)	0.0581 (8)	
H14	0.944487	0.542707	0.308477	0.070*	
C15	0.6987 (4)	0.5661 (3)	0.2515 (2)	0.0494 (6)	
H15	0.719222	0.661767	0.289891	0.059*	
C16	0.5265 (4)	0.4906 (3)	0.1804 (2)	0.0407 (5)	
C17	0.2954 (6)	0.1291 (3)	−0.0083 (3)	0.0663 (9)	
H17A	0.308435	0.055894	0.049069	0.099*	
H17B	0.384247	0.136518	−0.066635	0.099*	
H17C	0.169533	0.096781	−0.048532	0.099*	

### 4-Methylquinoline–2-chloro-5-nitrobenzoic acid (1/1) (II) Atomic displacement parameters (Å^2^)

**Table:**

	*U*^11^	*U*^22^	*U*^33^	*U*^12^	*U*^13^	*U*^23^
Cl1	0.0441 (4)	0.0620 (5)	0.0871 (6)	0.0058 (3)	−0.0173 (4)	−0.0221 (4)
O1	0.0728 (14)	0.0406 (10)	0.0539 (11)	0.0230 (9)	0.0045 (9)	−0.0123 (8)
O2	0.0988 (18)	0.0491 (12)	0.0566 (12)	0.0232 (12)	−0.0230 (12)	−0.0203 (10)
O3	0.0466 (10)	0.0364 (9)	0.0627 (12)	0.0073 (7)	−0.0089 (8)	−0.0114 (8)
O4	0.0359 (9)	0.0534 (11)	0.0668 (12)	0.0176 (8)	0.0023 (8)	−0.0033 (9)
N1	0.0352 (10)	0.0369 (10)	0.0432 (11)	0.0113 (8)	0.0030 (8)	−0.0016 (8)
N2	0.0537 (13)	0.0396 (11)	0.0455 (12)	0.0131 (9)	0.0075 (9)	−0.0053 (9)
C1	0.0492 (13)	0.0284 (10)	0.0344 (11)	0.0085 (9)	0.0017 (9)	−0.0028 (9)
C2	0.0384 (12)	0.0390 (12)	0.0450 (13)	0.0101 (9)	−0.0065 (10)	−0.0047 (10)
C3	0.0371 (12)	0.0416 (12)	0.0542 (14)	0.0138 (10)	−0.0017 (10)	−0.0087 (11)
C4	0.0356 (11)	0.0323 (11)	0.0463 (13)	0.0109 (9)	0.0009 (9)	−0.0079 (9)
C5	0.0390 (12)	0.0291 (10)	0.0338 (10)	0.0084 (8)	0.0017 (8)	−0.0009 (8)
C6	0.0429 (13)	0.0319 (11)	0.0358 (11)	0.0124 (9)	0.0061 (9)	−0.0006 (9)
C7	0.0654 (17)	0.0331 (11)	0.0380 (12)	0.0129 (11)	0.0035 (11)	−0.0028 (10)
C8	0.0437 (14)	0.0566 (16)	0.0452 (13)	0.0122 (12)	0.0048 (11)	0.0001 (12)
C9	0.0461 (14)	0.0491 (14)	0.0397 (12)	0.0094 (11)	−0.0028 (10)	−0.0008 (11)
C10	0.0552 (15)	0.0412 (13)	0.0337 (11)	0.0039 (11)	0.0039 (10)	−0.0050 (10)
C11	0.0413 (12)	0.0340 (11)	0.0300 (10)	0.0109 (9)	0.0050 (9)	−0.0012 (9)
C12	0.0601 (17)	0.0626 (18)	0.0518 (15)	0.0310 (14)	0.0171 (13)	0.0137 (14)
C13	0.0526 (17)	0.084 (2)	0.0628 (19)	0.0265 (16)	0.0152 (15)	0.0222 (18)
C14	0.0385 (14)	0.078 (2)	0.0502 (15)	0.0075 (13)	−0.0007 (11)	0.0121 (15)
C15	0.0504 (15)	0.0489 (14)	0.0374 (12)	−0.0006 (11)	0.0022 (10)	−0.0039 (11)
C16	0.0452 (13)	0.0404 (12)	0.0355 (11)	0.0112 (10)	0.0071 (9)	−0.0006 (10)
C17	0.092 (2)	0.0448 (15)	0.0506 (16)	0.0073 (15)	0.0014 (15)	−0.0109 (13)

### 4-Methylquinoline–2-chloro-5-nitrobenzoic acid (1/1) (II) Geometric parameters (Å, º)

**Table:**

Cl1—C2	1.723 (2)	C8—C9	1.380 (4)
O1—C7	1.310 (4)	C8—H8	0.9500
O1—H1	0.91 (7)	C9—C10	1.395 (4)
O2—C7	1.214 (3)	C9—H9	0.9500
O3—N1	1.224 (3)	C10—C11	1.440 (3)
O4—N1	1.235 (3)	C10—C17	1.481 (4)
N1—C5	1.462 (3)	C11—C12	1.397 (4)
N2—C8	1.313 (4)	C11—C16	1.423 (3)
N2—C16	1.356 (3)	C12—C13	1.333 (5)
C1—C6	1.397 (3)	C12—H12	0.9500
C1—C2	1.405 (4)	C13—C14	1.373 (5)
C1—C7	1.509 (3)	C13—H13	0.9500
C2—C3	1.383 (3)	C14—C15	1.372 (4)
C3—C4	1.380 (3)	C14—H14	0.9500
C3—H3	0.9500	C15—C16	1.446 (4)
C4—C5	1.380 (3)	C15—H15	0.9500
C4—H4	0.9500	C17—H17A	0.9800
C5—C6	1.383 (3)	C17—H17B	0.9800
C6—H6	0.9500	C17—H17C	0.9800
			
C7—O1—H1	102 (4)	C8—C9—H9	120.7
O3—N1—O4	123.8 (2)	C10—C9—H9	120.7
O3—N1—C5	118.12 (19)	C9—C10—C11	118.8 (2)
O4—N1—C5	118.0 (2)	C9—C10—C17	120.4 (3)
C8—N2—C16	118.2 (2)	C11—C10—C17	120.8 (3)
C6—C1—C2	118.0 (2)	C12—C11—C16	118.7 (2)
C6—C1—C7	117.9 (2)	C12—C11—C10	124.7 (2)
C2—C1—C7	124.1 (2)	C16—C11—C10	116.6 (2)
C3—C2—C1	121.4 (2)	C13—C12—C11	122.4 (3)
C3—C2—Cl1	115.90 (19)	C13—C12—H12	118.8
C1—C2—Cl1	122.63 (19)	C11—C12—H12	118.8
C4—C3—C2	120.3 (2)	C12—C13—C14	119.8 (3)
C4—C3—H3	119.9	C12—C13—H13	120.1
C2—C3—H3	119.9	C14—C13—H13	120.1
C5—C4—C3	118.4 (2)	C15—C14—C13	123.1 (3)
C5—C4—H4	120.8	C15—C14—H14	118.5
C3—C4—H4	120.8	C13—C14—H14	118.5
C4—C5—C6	122.6 (2)	C14—C15—C16	117.6 (3)
C4—C5—N1	118.62 (19)	C14—C15—H15	121.2
C6—C5—N1	118.8 (2)	C16—C15—H15	121.2
C5—C6—C1	119.3 (2)	N2—C16—C11	122.8 (2)
C5—C6—H6	120.4	N2—C16—C15	118.7 (2)
C1—C6—H6	120.4	C11—C16—C15	118.5 (2)
O2—C7—O1	125.0 (2)	C10—C17—H17A	109.5
O2—C7—C1	122.6 (3)	C10—C17—H17B	109.5
O1—C7—C1	112.4 (2)	H17A—C17—H17B	109.5
N2—C8—C9	125.2 (3)	C10—C17—H17C	109.5
N2—C8—H8	117.4	H17A—C17—H17C	109.5
C9—C8—H8	117.4	H17B—C17—H17C	109.5
C8—C9—C10	118.5 (2)		
			
C6—C1—C2—C3	1.9 (4)	C16—N2—C8—C9	−0.8 (4)
C7—C1—C2—C3	−178.0 (2)	N2—C8—C9—C10	0.6 (4)
C6—C1—C2—Cl1	−174.82 (18)	C8—C9—C10—C11	−0.3 (4)
C7—C1—C2—Cl1	5.2 (4)	C8—C9—C10—C17	−179.8 (3)
C1—C2—C3—C4	−0.2 (4)	C9—C10—C11—C12	−179.0 (2)
Cl1—C2—C3—C4	176.8 (2)	C17—C10—C11—C12	0.4 (4)
C2—C3—C4—C5	−1.6 (4)	C9—C10—C11—C16	0.4 (3)
C3—C4—C5—C6	1.6 (4)	C17—C10—C11—C16	179.8 (2)
C3—C4—C5—N1	−177.4 (2)	C16—C11—C12—C13	0.8 (4)
O3—N1—C5—C4	−8.7 (3)	C10—C11—C12—C13	−179.8 (3)
O4—N1—C5—C4	170.1 (2)	C11—C12—C13—C14	−0.8 (4)
O3—N1—C5—C6	172.3 (2)	C12—C13—C14—C15	0.1 (5)
O4—N1—C5—C6	−9.0 (3)	C13—C14—C15—C16	0.5 (4)
C4—C5—C6—C1	0.1 (4)	C8—N2—C16—C11	0.8 (4)
N1—C5—C6—C1	179.15 (19)	C8—N2—C16—C15	179.8 (2)
C2—C1—C6—C5	−1.9 (3)	C12—C11—C16—N2	178.8 (2)
C7—C1—C6—C5	178.1 (2)	C10—C11—C16—N2	−0.6 (3)
C6—C1—C7—O2	−154.9 (3)	C12—C11—C16—C15	−0.1 (3)
C2—C1—C7—O2	25.1 (4)	C10—C11—C16—C15	−179.6 (2)
C6—C1—C7—O1	23.9 (3)	C14—C15—C16—N2	−179.5 (2)
C2—C1—C7—O1	−156.2 (2)	C14—C15—C16—C11	−0.5 (4)

### 4-Methylquinoline–2-chloro-5-nitrobenzoic acid (1/1) (II) Hydrogen-bond geometry (Å, º)

**Table:**

*D*—H···*A*	*D*—H	H···*A*	*D*···*A*	*D*—H···*A*
O1—H1···N2	0.91 (7)	1.68 (7)	2.556 (3)	162 (7)
C3—H3···O4^i^	0.95	2.40	3.280 (4)	154
C4—H4···O3^ii^	0.95	2.54	3.188 (3)	126
C17—H17*A*···O2^iii^	0.98	2.57	3.479 (4)	155
C17—H17*C*···Cl1^iv^	0.98	2.81	3.535 (4)	131

Symmetry codes: (i) *x*−1, *y*, *z*; (ii) −*x*+1, −*y*+3, −*z*+1; (iii) *x*, *y*−1, *z*; (iv) −*x*, −*y*+1, −*z*.

### 4-Methylquinolinium 2-chloro-6-nitrobenzoate (III) Crystal data

**Table:**

C_10_H_9.63_N0.63+·C_7_H_3.37_ClNO_4_0.63−−	*F*(000) = 712.00
*M**_r_* = 344.75	*D*_x_ = 1.456 Mg m^−^^3^
Monoclinic, *P*2_1_/*c*	Mo *K*α radiation, λ = 0.71075 Å
*a* = 6.6401 (3) Å	Cell parameters from 25957 reflections
*b* = 23.2126 (5) Å	θ = 3.1–30.1°
*c* = 10.3386 (3) Å	µ = 0.27 mm^−^^1^
β = 99.3926 (15)°	*T* = 185 K
*V* = 1572.16 (9) Å^3^	Block, colorless
*Z* = 4	0.35 × 0.28 × 0.25 mm

### 4-Methylquinolinium 2-chloro-6-nitrobenzoate (III) Data collection

**Table:**

Rigaku R-AXIS RAPIDII diffractometer	3854 reflections with *I* > 2σ(*I*)
Detector resolution: 10.000 pixels mm^-1^	*R*_int_ = 0.022
ω scans	θ_max_ = 30.0°, θ_min_ = 3.1°
Absorption correction: numerical (NUMABS; Higashi, 1999)	*h* = −9→9
*T*_min_ = 0.909, *T*_max_ = 0.935	*k* = −32→32
32362 measured reflections	*l* = −14→14
4588 independent reflections	

### 4-Methylquinolinium 2-chloro-6-nitrobenzoate (III) Refinement

**Table:**

Refinement on *F*^2^	Secondary atom site location: difference Fourier map
*R*[*F*^2^ > 2σ(*F*^2^)] = 0.044	Hydrogen site location: mixed
*wR*(*F*^2^) = 0.125	H atoms treated by a mixture of independent and constrained refinement
*S* = 1.07	*w* = 1/[σ^2^(*F*_o_^2^) + (0.0685*P*)^2^ + 0.4164*P*] where *P* = (*F*_o_^2^ + 2*F*_c_^2^)/3
4588 reflections	(Δ/σ)_max_ = 0.001
244 parameters	Δρ_max_ = 0.52 e Å^−^^3^
2 restraints	Δρ_min_ = −0.40 e Å^−^^3^
Primary atom site location: structure-invariant direct methods	

### 4-Methylquinolinium 2-chloro-6-nitrobenzoate (III) Special details

**Table:**

Geometry. All esds (except the esd in the dihedral angle between two l.s. planes) are estimated using the full covariance matrix. The cell esds are taken into account individually in the estimation of esds in distances, angles and torsion angles; correlations between esds in cell parameters are only used when they are defined by crystal symmetry. An approximate (isotropic) treatment of cell esds is used for estimating esds involving l.s. planes.
Refinement. Refinement was performed using all reflections. The weighted R-factor (wR) and goodness of fit (S) are based on F^2^. R-factor (gt) are based on F. The threshold expression of F^2^ > 2.0 sigma(F^2^) is used only for calculating R-factor (gt).

### 4-Methylquinolinium 2-chloro-6-nitrobenzoate (III) Fractional atomic coordinates and isotropic or equivalent isotropic displacement parameters (Å^2^)

**Table:**

	*x*	*y*	*z*	*U*_iso_*/*U*_eq_	Occ. (<1)
Cl1	0.71027 (7)	0.10339 (2)	1.02427 (4)	0.05678 (15)	
O1	0.34983 (15)	0.13094 (4)	0.76226 (8)	0.0326 (2)	
H1	0.328 (8)	0.1007 (14)	0.717 (5)	0.049*	0.37 (3)
O2	0.18762 (19)	0.08197 (5)	0.89869 (10)	0.0468 (3)	
O3A	0.0115 (12)	0.2046 (4)	0.8201 (5)	0.0463 (13)	0.54 (3)
O4A	−0.0751 (8)	0.2537 (6)	0.9776 (7)	0.0623 (19)	0.54 (3)
O3B	−0.0248 (19)	0.1914 (7)	0.8468 (19)	0.078 (3)	0.46 (3)
O4B	−0.021 (3)	0.2723 (4)	0.9405 (16)	0.076 (4)	0.46 (3)
N1	0.04842 (19)	0.22525 (5)	0.92742 (12)	0.0376 (3)	
N2	0.28090 (14)	0.04202 (4)	0.61720 (9)	0.02414 (19)	
H2	0.300 (4)	0.0736 (7)	0.666 (2)	0.036*	0.63 (3)
C1	0.36395 (19)	0.16626 (5)	0.97746 (10)	0.0258 (2)	
C2	0.5557 (2)	0.15998 (6)	1.05519 (12)	0.0324 (3)	
C3	0.6290 (2)	0.19756 (7)	1.15678 (14)	0.0401 (3)	
H3	0.761107	0.192060	1.206500	0.048*	
C4	0.5092 (2)	0.24273 (6)	1.18481 (14)	0.0412 (3)	
H4	0.557720	0.268328	1.254673	0.049*	
C5	0.3182 (2)	0.25079 (5)	1.11112 (13)	0.0365 (3)	
H5	0.234047	0.281705	1.130255	0.044*	
C6	0.25041 (19)	0.21305 (5)	1.00842 (11)	0.0283 (2)	
C7	0.28934 (19)	0.12224 (5)	0.87118 (11)	0.0266 (2)	
C8	0.28861 (17)	−0.00796 (5)	0.67783 (11)	0.0266 (2)	
H8	0.308842	−0.008856	0.770917	0.032*	
C9	0.26769 (18)	−0.05958 (5)	0.60825 (12)	0.0283 (2)	
H9	0.273935	−0.095108	0.654330	0.034*	
C10	0.23797 (17)	−0.05958 (5)	0.47294 (12)	0.0272 (2)	
C11	0.22837 (16)	−0.00558 (5)	0.40662 (11)	0.0244 (2)	
C12	0.19706 (19)	0.00027 (6)	0.26760 (12)	0.0335 (3)	
H12	0.180685	−0.033200	0.214019	0.040*	
C13	0.1903 (2)	0.05339 (7)	0.21055 (13)	0.0395 (3)	
H13	0.169884	0.056667	0.117731	0.047*	
C14	0.2134 (2)	0.10307 (6)	0.28825 (14)	0.0373 (3)	
H14	0.208084	0.139770	0.247145	0.045*	
C15	0.24365 (18)	0.09990 (5)	0.42260 (13)	0.0301 (2)	
H15	0.258987	0.133966	0.474193	0.036*	
C16	0.25156 (16)	0.04526 (5)	0.48282 (11)	0.0233 (2)	
C17	0.2150 (2)	−0.11468 (6)	0.39687 (16)	0.0402 (3)	
H17A	0.081819	−0.115295	0.339800	0.060*	
H17B	0.224727	−0.147324	0.457681	0.060*	
H17C	0.323533	−0.117515	0.343293	0.060*	

### 4-Methylquinolinium 2-chloro-6-nitrobenzoate (III) Atomic displacement parameters (Å^2^)

**Table:**

	*U*^11^	*U*^22^	*U*^33^	*U*^12^	*U*^13^	*U*^23^
Cl1	0.0537 (2)	0.0714 (3)	0.0417 (2)	0.0315 (2)	−0.00294 (16)	−0.00849 (18)
O1	0.0511 (5)	0.0267 (4)	0.0208 (4)	−0.0049 (4)	0.0088 (4)	−0.0020 (3)
O2	0.0706 (7)	0.0442 (5)	0.0286 (5)	−0.0253 (5)	0.0173 (5)	−0.0089 (4)
O3A	0.041 (2)	0.064 (3)	0.0298 (17)	0.0180 (18)	−0.0048 (11)	−0.0085 (14)
O4A	0.0501 (18)	0.068 (4)	0.069 (2)	0.0233 (19)	0.0096 (17)	−0.023 (2)
O3B	0.047 (3)	0.086 (6)	0.092 (6)	0.014 (3)	−0.020 (4)	−0.050 (5)
O4B	0.076 (5)	0.048 (3)	0.088 (5)	0.032 (3)	−0.031 (4)	−0.021 (3)
N1	0.0447 (6)	0.0327 (5)	0.0347 (6)	0.0086 (5)	0.0043 (5)	−0.0038 (4)
N2	0.0241 (4)	0.0274 (4)	0.0211 (4)	0.0004 (3)	0.0041 (3)	−0.0024 (3)
C1	0.0332 (6)	0.0250 (5)	0.0191 (5)	−0.0005 (4)	0.0040 (4)	−0.0011 (4)
C2	0.0344 (6)	0.0360 (6)	0.0259 (5)	0.0040 (5)	0.0020 (4)	−0.0015 (5)
C3	0.0396 (7)	0.0467 (7)	0.0306 (6)	−0.0056 (6)	−0.0040 (5)	−0.0030 (6)
C4	0.0567 (9)	0.0334 (6)	0.0308 (6)	−0.0102 (6)	−0.0011 (6)	−0.0079 (5)
C5	0.0545 (8)	0.0237 (5)	0.0308 (6)	0.0003 (5)	0.0052 (5)	−0.0048 (5)
C6	0.0365 (6)	0.0240 (5)	0.0236 (5)	0.0005 (4)	0.0030 (4)	−0.0012 (4)
C7	0.0343 (6)	0.0249 (5)	0.0202 (5)	0.0015 (4)	0.0033 (4)	−0.0026 (4)
C8	0.0253 (5)	0.0323 (6)	0.0225 (5)	0.0015 (4)	0.0050 (4)	0.0023 (4)
C9	0.0253 (5)	0.0272 (5)	0.0330 (6)	0.0007 (4)	0.0068 (4)	0.0035 (4)
C10	0.0214 (5)	0.0273 (5)	0.0340 (6)	−0.0012 (4)	0.0073 (4)	−0.0056 (4)
C11	0.0191 (4)	0.0316 (5)	0.0229 (5)	−0.0006 (4)	0.0042 (4)	−0.0040 (4)
C12	0.0277 (6)	0.0504 (7)	0.0227 (5)	−0.0010 (5)	0.0044 (4)	−0.0072 (5)
C13	0.0325 (6)	0.0636 (9)	0.0226 (5)	0.0020 (6)	0.0047 (5)	0.0072 (6)
C14	0.0334 (6)	0.0450 (7)	0.0343 (6)	0.0041 (5)	0.0076 (5)	0.0147 (5)
C15	0.0288 (5)	0.0300 (6)	0.0321 (6)	0.0010 (4)	0.0063 (4)	0.0043 (4)
C16	0.0200 (5)	0.0281 (5)	0.0222 (5)	0.0003 (4)	0.0044 (4)	−0.0009 (4)
C17	0.0390 (7)	0.0321 (6)	0.0505 (8)	−0.0048 (5)	0.0106 (6)	−0.0155 (6)

### 4-Methylquinolinium 2-chloro-6-nitrobenzoate (III) Geometric parameters (Å, º)

**Table:**

Cl1—C2	1.7288 (13)	C5—H5	0.9500
O1—C7	1.2720 (14)	C8—C9	1.3928 (17)
O1—H1	0.841 (10)	C8—H8	0.9500
O2—C7	1.2140 (16)	C9—C10	1.3805 (17)
O3A—N1	1.196 (7)	C9—H9	0.9500
O4A—N1	1.232 (4)	C10—C11	1.4251 (16)
O3B—N1	1.190 (9)	C10—C17	1.4961 (17)
O4B—N1	1.201 (5)	C11—C16	1.4134 (15)
N1—C6	1.4879 (17)	C11—C12	1.4248 (16)
N2—C8	1.3157 (15)	C12—C13	1.365 (2)
N2—C16	1.3731 (14)	C12—H12	0.9500
N2—H2	0.887 (10)	C13—C14	1.399 (2)
C1—C6	1.3888 (16)	C13—H13	0.9500
C1—C2	1.3978 (17)	C14—C15	1.3726 (18)
C1—C7	1.5229 (15)	C14—H14	0.9500
C2—C3	1.3908 (19)	C15—C16	1.4100 (16)
C3—C4	1.375 (2)	C15—H15	0.9500
C3—H3	0.9500	C17—H17A	0.9800
C4—C5	1.382 (2)	C17—H17B	0.9800
C4—H4	0.9500	C17—H17C	0.9800
C5—C6	1.3934 (17)		
			
C7—O1—H1	108 (4)	N2—C8—H8	119.3
O3B—N1—O4B	124.0 (7)	C9—C8—H8	119.3
O3A—N1—O4A	123.7 (4)	C10—C9—C8	120.61 (11)
O3B—N1—C6	119.8 (5)	C10—C9—H9	119.7
O3A—N1—C6	118.4 (3)	C8—C9—H9	119.7
O4B—N1—C6	115.8 (4)	C9—C10—C11	118.37 (10)
O4A—N1—C6	117.8 (3)	C9—C10—C17	121.21 (12)
C8—N2—C16	121.22 (10)	C11—C10—C17	120.42 (12)
C8—N2—H2	117.8 (18)	C16—C11—C12	117.86 (11)
C16—N2—H2	120.9 (18)	C16—C11—C10	118.30 (10)
C6—C1—C2	115.27 (10)	C12—C11—C10	123.84 (11)
C6—C1—C7	124.47 (11)	C13—C12—C11	120.75 (12)
C2—C1—C7	120.22 (10)	C13—C12—H12	119.6
C3—C2—C1	122.86 (12)	C11—C12—H12	119.6
C3—C2—Cl1	118.09 (11)	C12—C13—C14	120.25 (12)
C1—C2—Cl1	119.05 (9)	C12—C13—H13	119.9
C4—C3—C2	119.60 (13)	C14—C13—H13	119.9
C4—C3—H3	120.2	C15—C14—C13	121.39 (12)
C2—C3—H3	120.2	C15—C14—H14	119.3
C3—C4—C5	119.84 (12)	C13—C14—H14	119.3
C3—C4—H4	120.1	C14—C15—C16	118.94 (12)
C5—C4—H4	120.1	C14—C15—H15	120.5
C4—C5—C6	119.24 (13)	C16—C15—H15	120.5
C4—C5—H5	120.4	N2—C16—C15	119.01 (10)
C6—C5—H5	120.4	N2—C16—C11	120.18 (10)
C1—C6—C5	123.18 (12)	C15—C16—C11	120.81 (10)
C1—C6—N1	119.51 (10)	C10—C17—H17A	109.5
C5—C6—N1	117.30 (11)	C10—C17—H17B	109.5
O2—C7—O1	126.71 (11)	H17A—C17—H17B	109.5
O2—C7—C1	118.40 (10)	C10—C17—H17C	109.5
O1—C7—C1	114.82 (10)	H17A—C17—H17C	109.5
N2—C8—C9	121.31 (10)	H17B—C17—H17C	109.5
			
C6—C1—C2—C3	0.13 (19)	C6—C1—C7—O1	98.00 (14)
C7—C1—C2—C3	−177.61 (12)	C2—C1—C7—O1	−84.49 (14)
C6—C1—C2—Cl1	−179.59 (9)	C16—N2—C8—C9	0.11 (17)
C7—C1—C2—Cl1	2.68 (16)	N2—C8—C9—C10	−0.05 (17)
C1—C2—C3—C4	0.9 (2)	C8—C9—C10—C11	−0.28 (17)
Cl1—C2—C3—C4	−179.40 (12)	C8—C9—C10—C17	−179.94 (11)
C2—C3—C4—C5	−0.7 (2)	C9—C10—C11—C16	0.54 (16)
C3—C4—C5—C6	−0.5 (2)	C17—C10—C11—C16	−179.80 (11)
C2—C1—C6—C5	−1.36 (18)	C9—C10—C11—C12	−179.58 (11)
C7—C1—C6—C5	176.26 (12)	C17—C10—C11—C12	0.08 (17)
C2—C1—C6—N1	177.02 (11)	C16—C11—C12—C13	0.15 (17)
C7—C1—C6—N1	−5.35 (18)	C10—C11—C12—C13	−179.73 (11)
C4—C5—C6—C1	1.6 (2)	C11—C12—C13—C14	−0.2 (2)
C4—C5—C6—N1	−176.84 (12)	C12—C13—C14—C15	0.1 (2)
O3B—N1—C6—C1	8.6 (14)	C13—C14—C15—C16	0.05 (19)
O3A—N1—C6—C1	−19.0 (5)	C8—N2—C16—C15	179.75 (10)
O4B—N1—C6—C1	−164.4 (14)	C8—N2—C16—C11	0.17 (16)
O4A—N1—C6—C1	157.3 (8)	C14—C15—C16—N2	−179.72 (11)
O3B—N1—C6—C5	−172.9 (14)	C14—C15—C16—C11	−0.15 (18)
O3A—N1—C6—C5	159.4 (5)	C12—C11—C16—N2	179.62 (10)
O4B—N1—C6—C5	14.1 (14)	C10—C11—C16—N2	−0.49 (16)
O4A—N1—C6—C5	−24.2 (8)	C12—C11—C16—C15	0.05 (16)
C6—C1—C7—O2	−84.88 (16)	C10—C11—C16—C15	179.94 (10)
C2—C1—C7—O2	92.63 (16)		

### 4-Methylquinolinium 2-chloro-6-nitrobenzoate (III) Hydrogen-bond geometry (Å, º)

*Cg*1 is the centroid of the C1–C6 ring.

**Table:**

*D*—H···*A*	*D*—H	H···*A*	*D*···*A*	*D*—H···*A*
O1—H1···N2	0.84 (4)	1.71 (4)	2.5485 (13)	177 (6)
N2—H2···O1	0.89 (2)	1.66 (2)	2.5485 (13)	176 (2)
C5—H5···O1^i^	0.95	2.49	3.1489 (15)	126
C13—H13···O2^ii^	0.95	2.36	3.2889 (17)	165
C14—H14···*Cg*1^ii^	0.95	2.89	3.6596 (15)	138

Symmetry codes: (i) *x*, −*y*+1/2, *z*+1/2; (ii) *x*, *y*, *z*−1.

### 4-Methylquinolinium 3-chloro-2-nitrobenzoate (IV) Crystal data

**Table:**

C_10_H_9.54_N0.54+·C_7_H_3.46_ClNO_4_0.54−−	*Z* = 2
*M**_r_* = 344.75	*F*(000) = 356.00
Triclinic, *P*1	*D*_x_ = 1.470 Mg m^−^^3^
*a* = 7.5234 (10) Å	Mo *K*α radiation, λ = 0.71075 Å
*b* = 7.8017 (11) Å	Cell parameters from 14620 reflections
*c* = 13.6341 (17) Å	θ = 3.1–30.2°
α = 80.934 (4)°	µ = 0.27 mm^−^^1^
β = 80.227 (3)°	*T* = 185 K
γ = 89.150 (4)°	Block, colorless
*V* = 778.73 (18) Å^3^	0.35 × 0.29 × 0.22 mm

### 4-Methylquinolinium 3-chloro-2-nitrobenzoate (IV) Data collection

**Table:**

Rigaku R-AXIS RAPIDII diffractometer	4017 reflections with *I* > 2σ(*I*)
Detector resolution: 10.000 pixels mm^-1^	*R*_int_ = 0.028
ω scans	θ_max_ = 30.0°, θ_min_ = 3.1°
Absorption correction: numerical (NUMABS; Higashi, 1999)	*h* = −10→10
*T*_min_ = 0.914, *T*_max_ = 0.942	*k* = −10→10
16767 measured reflections	*l* = −19→19
4544 independent reflections	

### 4-Methylquinolinium 3-chloro-2-nitrobenzoate (IV) Refinement

**Table:**

Refinement on *F*^2^	Secondary atom site location: difference Fourier map
*R*[*F*^2^ > 2σ(*F*^2^)] = 0.036	Hydrogen site location: inferred from neighbouring sites
*wR*(*F*^2^) = 0.103	H atoms treated by a mixture of independent and constrained refinement
*S* = 1.07	*w* = 1/[σ^2^(*F*_o_^2^) + (0.0589*P*)^2^ + 0.1388*P*] where *P* = (*F*_o_^2^ + 2*F*_c_^2^)/3
4544 reflections	(Δ/σ)_max_ = 0.001
225 parameters	Δρ_max_ = 0.44 e Å^−^^3^
2 restraints	Δρ_min_ = −0.38 e Å^−^^3^
Primary atom site location: structure-invariant direct methods	

### 4-Methylquinolinium 3-chloro-2-nitrobenzoate (IV) Special details

**Table:**

Geometry. All esds (except the esd in the dihedral angle between two l.s. planes) are estimated using the full covariance matrix. The cell esds are taken into account individually in the estimation of esds in distances, angles and torsion angles; correlations between esds in cell parameters are only used when they are defined by crystal symmetry. An approximate (isotropic) treatment of cell esds is used for estimating esds involving l.s. planes.

### 4-Methylquinolinium 3-chloro-2-nitrobenzoate (IV) Fractional atomic coordinates and isotropic or equivalent isotropic displacement parameters (Å^2^)

**Table:**

	*x*	*y*	*z*	*U*_iso_*/*U*_eq_	Occ. (<1)
Cl1	0.86435 (3)	0.88078 (4)	−0.07310 (2)	0.03700 (9)	
O1	0.32371 (11)	0.40966 (11)	0.26115 (7)	0.0398 (2)	
H1	0.318 (6)	0.339 (4)	0.3152 (18)	0.060*	0.46 (3)
O2	0.56467 (12)	0.53822 (11)	0.29489 (6)	0.03676 (18)	
O3	0.87768 (12)	0.52646 (12)	0.12927 (8)	0.0450 (2)	
O4	0.86170 (12)	0.78937 (12)	0.16317 (6)	0.0410 (2)	
N1	0.80578 (11)	0.66730 (12)	0.13027 (7)	0.02921 (18)	
N2	0.30583 (11)	0.21139 (11)	0.42899 (7)	0.02971 (18)	
H2	0.318 (4)	0.278 (3)	0.3693 (12)	0.045*	0.54 (3)
C1	0.47878 (12)	0.61791 (12)	0.13339 (7)	0.02463 (18)	
C2	0.64206 (12)	0.69496 (12)	0.08391 (7)	0.02335 (18)	
C3	0.65928 (13)	0.79149 (12)	−0.01201 (7)	0.02544 (18)	
C4	0.51016 (14)	0.81598 (14)	−0.06009 (8)	0.0305 (2)	
H4	0.520225	0.883772	−0.125138	0.037*	
C5	0.34671 (14)	0.74038 (14)	−0.01204 (9)	0.0318 (2)	
H5	0.244061	0.756140	−0.044445	0.038*	
C6	0.33155 (13)	0.64142 (13)	0.08340 (8)	0.0288 (2)	
H6	0.218788	0.589069	0.114930	0.035*	
C7	0.45882 (13)	0.51586 (13)	0.23876 (8)	0.02791 (19)	
C8	0.34235 (14)	0.26031 (14)	0.51192 (9)	0.0334 (2)	
H8	0.382978	0.376070	0.508800	0.040*	
C9	0.32371 (15)	0.14881 (15)	0.60416 (9)	0.0336 (2)	
H9	0.350523	0.189658	0.662139	0.040*	
C10	0.26665 (13)	−0.01971 (14)	0.61092 (8)	0.0293 (2)	
C11	0.23111 (12)	−0.07674 (12)	0.52147 (7)	0.02621 (19)	
C12	0.17762 (15)	−0.24889 (14)	0.51777 (9)	0.0347 (2)	
H12	0.163822	−0.333039	0.577111	0.042*	
C13	0.14562 (17)	−0.29488 (16)	0.42911 (10)	0.0403 (3)	
H13	0.110950	−0.410964	0.427749	0.048*	
C14	0.16351 (16)	−0.17203 (17)	0.33985 (10)	0.0395 (3)	
H14	0.139455	−0.205611	0.279328	0.047*	
C15	0.21525 (14)	−0.00529 (15)	0.34041 (8)	0.0329 (2)	
H15	0.227352	0.077170	0.280383	0.039*	
C16	0.25086 (12)	0.04439 (13)	0.43088 (7)	0.02605 (19)	
C17	0.24210 (18)	−0.14002 (17)	0.70976 (9)	0.0420 (3)	
H17A	0.116898	−0.182138	0.727282	0.063*	
H17B	0.270185	−0.077702	0.762387	0.063*	
H17C	0.323199	−0.238764	0.704128	0.063*	

### 4-Methylquinolinium 3-chloro-2-nitrobenzoate (IV) Atomic displacement parameters (Å^2^)

**Table:**

	*U*^11^	*U*^22^	*U*^33^	*U*^12^	*U*^13^	*U*^23^
Cl1	0.02892 (13)	0.04837 (17)	0.02927 (14)	−0.01052 (10)	0.00070 (10)	0.00234 (10)
O1	0.0339 (4)	0.0411 (4)	0.0395 (4)	−0.0140 (3)	−0.0068 (3)	0.0110 (3)
O2	0.0414 (4)	0.0382 (4)	0.0302 (4)	−0.0095 (3)	−0.0086 (3)	0.0001 (3)
O3	0.0310 (4)	0.0465 (5)	0.0587 (6)	0.0088 (3)	−0.0142 (4)	−0.0051 (4)
O4	0.0398 (4)	0.0503 (5)	0.0349 (4)	−0.0183 (4)	−0.0104 (3)	−0.0065 (3)
N1	0.0232 (4)	0.0370 (4)	0.0265 (4)	−0.0061 (3)	−0.0045 (3)	−0.0013 (3)
N2	0.0233 (4)	0.0298 (4)	0.0324 (4)	−0.0037 (3)	−0.0010 (3)	0.0027 (3)
C1	0.0232 (4)	0.0226 (4)	0.0273 (4)	−0.0020 (3)	−0.0026 (3)	−0.0029 (3)
C2	0.0211 (4)	0.0241 (4)	0.0254 (4)	−0.0013 (3)	−0.0045 (3)	−0.0049 (3)
C3	0.0240 (4)	0.0259 (4)	0.0255 (4)	−0.0036 (3)	−0.0013 (3)	−0.0040 (3)
C4	0.0318 (5)	0.0327 (5)	0.0265 (4)	0.0001 (4)	−0.0067 (4)	−0.0010 (4)
C5	0.0264 (5)	0.0354 (5)	0.0350 (5)	0.0005 (4)	−0.0103 (4)	−0.0041 (4)
C6	0.0219 (4)	0.0291 (5)	0.0350 (5)	−0.0025 (3)	−0.0043 (4)	−0.0038 (4)
C7	0.0263 (4)	0.0258 (4)	0.0292 (5)	−0.0017 (3)	−0.0010 (4)	−0.0009 (3)
C8	0.0259 (5)	0.0310 (5)	0.0428 (6)	−0.0037 (4)	−0.0042 (4)	−0.0056 (4)
C9	0.0287 (5)	0.0403 (6)	0.0339 (5)	0.0020 (4)	−0.0074 (4)	−0.0103 (4)
C10	0.0244 (4)	0.0352 (5)	0.0261 (4)	0.0056 (4)	−0.0021 (4)	−0.0015 (4)
C11	0.0211 (4)	0.0279 (4)	0.0269 (4)	0.0010 (3)	0.0004 (3)	−0.0007 (3)
C12	0.0340 (5)	0.0270 (5)	0.0388 (6)	−0.0009 (4)	0.0016 (4)	−0.0004 (4)
C13	0.0370 (6)	0.0336 (5)	0.0506 (7)	−0.0034 (4)	−0.0027 (5)	−0.0126 (5)
C14	0.0342 (5)	0.0488 (7)	0.0385 (6)	0.0007 (5)	−0.0064 (5)	−0.0164 (5)
C15	0.0286 (5)	0.0429 (6)	0.0259 (5)	0.0003 (4)	−0.0032 (4)	−0.0029 (4)
C16	0.0190 (4)	0.0299 (5)	0.0268 (4)	−0.0007 (3)	−0.0002 (3)	−0.0009 (3)
C17	0.0474 (7)	0.0469 (7)	0.0275 (5)	0.0104 (5)	−0.0033 (5)	0.0028 (4)

### 4-Methylquinolinium 3-chloro-2-nitrobenzoate (IV) Geometric parameters (Å, º)

**Table:**

Cl1—C3	1.7240 (10)	C8—C9	1.3990 (16)
O1—C7	1.2857 (12)	C8—H8	0.9500
O1—H1	0.843 (10)	C9—C10	1.3735 (16)
O2—C7	1.2261 (13)	C9—H9	0.9500
O3—N1	1.2185 (13)	C10—C11	1.4289 (14)
O4—N1	1.2230 (12)	C10—C17	1.5010 (15)
N1—C2	1.4753 (12)	C11—C12	1.4186 (14)
N2—C8	1.3206 (15)	C11—C16	1.4194 (13)
N2—C16	1.3678 (13)	C12—C13	1.3733 (18)
N2—H2	0.885 (10)	C12—H12	0.9500
C1—C6	1.3913 (14)	C13—C14	1.4137 (19)
C1—C2	1.3922 (13)	C13—H13	0.9500
C1—C7	1.5134 (14)	C14—C15	1.3649 (17)
C2—C3	1.3891 (13)	C14—H14	0.9500
C3—C4	1.3887 (14)	C15—C16	1.4176 (14)
C4—C5	1.3840 (15)	C15—H15	0.9500
C4—H4	0.9500	C17—H17A	0.9800
C5—C6	1.3921 (15)	C17—H17B	0.9800
C5—H5	0.9500	C17—H17C	0.9800
C6—H6	0.9500		
			
C7—O1—H1	116 (3)	C10—C9—C8	120.01 (10)
O3—N1—O4	125.42 (10)	C10—C9—H9	120.0
O3—N1—C2	117.11 (9)	C8—C9—H9	120.0
O4—N1—C2	117.42 (9)	C9—C10—C11	118.16 (9)
C8—N2—C16	119.82 (9)	C9—C10—C17	120.72 (10)
C8—N2—H2	125 (2)	C11—C10—C17	121.12 (10)
C16—N2—H2	115 (2)	C12—C11—C16	117.78 (9)
C6—C1—C2	117.57 (9)	C12—C11—C10	123.60 (9)
C6—C1—C7	120.60 (8)	C16—C11—C10	118.62 (9)
C2—C1—C7	121.81 (8)	C13—C12—C11	120.54 (10)
C3—C2—C1	121.74 (9)	C13—C12—H12	119.7
C3—C2—N1	117.82 (8)	C11—C12—H12	119.7
C1—C2—N1	120.35 (8)	C12—C13—C14	120.97 (11)
C4—C3—C2	119.89 (9)	C12—C13—H13	119.5
C4—C3—Cl1	119.23 (8)	C14—C13—H13	119.5
C2—C3—Cl1	120.88 (7)	C15—C14—C13	120.16 (11)
C5—C4—C3	119.16 (9)	C15—C14—H14	119.9
C5—C4—H4	120.4	C13—C14—H14	119.9
C3—C4—H4	120.4	C14—C15—C16	119.76 (10)
C4—C5—C6	120.51 (9)	C14—C15—H15	120.1
C4—C5—H5	119.7	C16—C15—H15	120.1
C6—C5—H5	119.7	N2—C16—C15	118.62 (9)
C1—C6—C5	121.11 (9)	N2—C16—C11	120.61 (9)
C1—C6—H6	119.4	C15—C16—C11	120.78 (9)
C5—C6—H6	119.4	C10—C17—H17A	109.5
O2—C7—O1	125.49 (10)	C10—C17—H17B	109.5
O2—C7—C1	120.78 (9)	H17A—C17—H17B	109.5
O1—C7—C1	113.73 (9)	C10—C17—H17C	109.5
N2—C8—C9	122.75 (10)	H17A—C17—H17C	109.5
N2—C8—H8	118.6	H17B—C17—H17C	109.5
C9—C8—H8	118.6		
			
C6—C1—C2—C3	−0.26 (14)	C16—N2—C8—C9	−1.42 (16)
C7—C1—C2—C3	−178.70 (9)	N2—C8—C9—C10	0.53 (17)
C6—C1—C2—N1	−176.66 (9)	C8—C9—C10—C11	1.18 (15)
C7—C1—C2—N1	4.90 (14)	C8—C9—C10—C17	−178.68 (10)
O3—N1—C2—C3	−101.75 (11)	C9—C10—C11—C12	177.75 (10)
O4—N1—C2—C3	75.67 (12)	C17—C10—C11—C12	−2.39 (15)
O3—N1—C2—C1	74.79 (12)	C9—C10—C11—C16	−1.95 (14)
O4—N1—C2—C1	−107.78 (11)	C17—C10—C11—C16	177.91 (9)
C1—C2—C3—C4	1.32 (15)	C16—C11—C12—C13	−0.30 (15)
N1—C2—C3—C4	177.81 (9)	C10—C11—C12—C13	180.00 (10)
C1—C2—C3—Cl1	−178.25 (7)	C11—C12—C13—C14	−0.57 (18)
N1—C2—C3—Cl1	−1.76 (12)	C12—C13—C14—C15	0.76 (18)
C2—C3—C4—C5	−1.27 (15)	C13—C14—C15—C16	−0.06 (17)
Cl1—C3—C4—C5	178.31 (8)	C8—N2—C16—C15	−179.04 (9)
C3—C4—C5—C6	0.19 (16)	C8—N2—C16—C11	0.56 (14)
C2—C1—C6—C5	−0.84 (15)	C14—C15—C16—N2	178.77 (10)
C7—C1—C6—C5	177.62 (9)	C14—C15—C16—C11	−0.82 (15)
C4—C5—C6—C1	0.89 (16)	C12—C11—C16—N2	−178.59 (9)
C6—C1—C7—O2	−156.92 (10)	C10—C11—C16—N2	1.12 (14)
C2—C1—C7—O2	21.48 (15)	C12—C11—C16—C15	0.99 (14)
C6—C1—C7—O1	22.59 (14)	C10—C11—C16—C15	−179.29 (9)
C2—C1—C7—O1	−159.02 (10)		

### 4-Methylquinolinium 3-chloro-2-nitrobenzoate (IV) Hydrogen-bond geometry (Å, º)

**Table:**

*D*—H···*A*	*D*—H	H···*A*	*D*···*A*	*D*—H···*A*
O1—H1···N2	0.84 (3)	1.70 (3)	2.5364 (13)	175 (3)
N2—H2···O1	0.89 (2)	1.65 (2)	2.5364 (13)	175 (3)
C6—H6···O3^i^	0.95	2.59	3.4705 (14)	155
C9—H9···O2^ii^	0.95	2.41	3.1739 (15)	137
C17—H17*C*···O2^iii^	0.98	2.47	3.4155 (17)	162

Symmetry codes: (i) *x*−1, *y*, *z*; (ii) −*x*+1, −*y*+1, −*z*+1; (iii) −*x*+1, −*y*, −*z*+1.

### 4-Methylquinolinium 4-chloro-2-nitrobenzoate (V) Crystal data

**Table:**

C_10_H_10_N^+^·C_7_H_3_ClNO_4_^−^	*Z* = 2
*M**_r_* = 344.75	*F*(000) = 356.00
Triclinic, *P*1	*D*_x_ = 1.469 Mg m^−^^3^
*a* = 7.6858 (3) Å	Mo *K*α radiation, λ = 0.71075 Å
*b* = 8.3615 (3) Å	Cell parameters from 19686 reflections
*c* = 13.5746 (5) Å	θ = 3.0–30.1°
α = 82.5485 (13)°	µ = 0.27 mm^−^^1^
β = 80.8927 (12)°	*T* = 185 K
γ = 65.0929 (11)°	Platelet, colorless
*V* = 779.33 (5) Å^3^	0.51 × 0.45 × 0.15 mm

### 4-Methylquinolinium 4-chloro-2-nitrobenzoate (V) Data collection

**Table:**

Rigaku R-AXIS RAPIDII diffractometer	3290 reflections with *I* > 2σ(*I*)
Detector resolution: 10.000 pixels mm^-1^	*R*_int_ = 0.027
ω scans	θ_max_ = 27.5°, θ_min_ = 3.1°
Absorption correction: numerical (NUMABS; Higashi, 1999)	*h* = −9→9
*T*_min_ = 0.868, *T*_max_ = 0.960	*k* = −10→10
18635 measured reflections	*l* = −17→17
3566 independent reflections	

### 4-Methylquinolinium 4-chloro-2-nitrobenzoate (V) Refinement

**Table:**

Refinement on *F*^2^	Secondary atom site location: difference Fourier map
*R*[*F*^2^ > 2σ(*F*^2^)] = 0.036	Hydrogen site location: mixed
*wR*(*F*^2^) = 0.102	H atoms treated by a mixture of independent and constrained refinement
*S* = 1.04	*w* = 1/[σ^2^(*F*_o_^2^) + (0.0588*P*)^2^ + 0.2103*P*] where *P* = (*F*_o_^2^ + 2*F*_c_^2^)/3
3566 reflections	(Δ/σ)_max_ = 0.002
222 parameters	Δρ_max_ = 0.38 e Å^−^^3^
0 restraints	Δρ_min_ = −0.18 e Å^−^^3^
Primary atom site location: structure-invariant direct methods	

### 4-Methylquinolinium 4-chloro-2-nitrobenzoate (V) Special details

**Table:**

Geometry. All esds (except the esd in the dihedral angle between two l.s. planes) are estimated using the full covariance matrix. The cell esds are taken into account individually in the estimation of esds in distances, angles and torsion angles; correlations between esds in cell parameters are only used when they are defined by crystal symmetry. An approximate (isotropic) treatment of cell esds is used for estimating esds involving l.s. planes.

### 4-Methylquinolinium 4-chloro-2-nitrobenzoate (V) Fractional atomic coordinates and isotropic or equivalent isotropic displacement parameters (Å^2^)

**Table:**

	*x*	*y*	*z*	*U*_iso_*/*U*_eq_	
Cl1	0.68899 (5)	−0.41758 (5)	1.09613 (2)	0.04437 (12)	
O1	0.55730 (12)	0.11536 (12)	0.67899 (7)	0.0343 (2)	
O2	0.27487 (13)	0.08937 (13)	0.70351 (7)	0.0361 (2)	
O3	0.04742 (14)	0.09528 (15)	0.90266 (8)	0.0491 (3)	
O4	0.12351 (16)	−0.15067 (16)	0.83652 (9)	0.0518 (3)	
N1	0.16299 (14)	−0.04644 (15)	0.87379 (8)	0.0336 (2)	
N2	0.42785 (14)	0.30577 (13)	0.52175 (7)	0.0270 (2)	
H2	0.483 (3)	0.226 (3)	0.5866 (16)	0.075 (6)*	
C1	0.49025 (15)	−0.04440 (14)	0.82541 (8)	0.0237 (2)	
C2	0.36650 (16)	−0.10303 (15)	0.89062 (8)	0.0255 (2)	
C3	0.42201 (17)	−0.21590 (16)	0.97438 (9)	0.0291 (2)	
H3	0.334084	−0.253809	1.017135	0.035*	
C4	0.61139 (17)	−0.27175 (16)	0.99359 (9)	0.0289 (2)	
C5	0.73961 (17)	−0.21407 (16)	0.93296 (9)	0.0294 (2)	
H5	0.868057	−0.251966	0.948261	0.035*	
C6	0.67764 (16)	−0.10049 (15)	0.84981 (9)	0.0271 (2)	
H6	0.764664	−0.059768	0.808401	0.033*	
C7	0.43193 (16)	0.06384 (15)	0.72882 (8)	0.0261 (2)	
C8	0.50883 (17)	0.25320 (16)	0.43209 (9)	0.0304 (2)	
H8	0.615416	0.141673	0.426068	0.036*	
C9	0.44242 (18)	0.35661 (17)	0.34601 (9)	0.0314 (3)	
H9	0.504996	0.315398	0.282367	0.038*	
C10	0.28780 (17)	0.51732 (16)	0.35173 (9)	0.0291 (2)	
C11	0.19978 (16)	0.57586 (15)	0.44844 (9)	0.0264 (2)	
C12	0.04213 (18)	0.74036 (17)	0.46435 (11)	0.0350 (3)	
H12	−0.011398	0.817344	0.408744	0.042*	
C13	−0.03350 (19)	0.78908 (18)	0.55913 (12)	0.0400 (3)	
H13	−0.138711	0.900418	0.568996	0.048*	
C14	0.0424 (2)	0.67651 (19)	0.64235 (10)	0.0393 (3)	
H14	−0.012424	0.712464	0.707852	0.047*	
C15	0.19339 (18)	0.51667 (17)	0.63033 (9)	0.0327 (3)	
H15	0.243434	0.440717	0.686908	0.039*	
C16	0.27446 (16)	0.46528 (15)	0.53306 (8)	0.0253 (2)	
C17	0.2148 (2)	0.6286 (2)	0.25939 (10)	0.0405 (3)	
H17A	0.075011	0.665044	0.263552	0.061*	
H17B	0.278616	0.560052	0.200744	0.061*	
H17C	0.243044	0.733558	0.253298	0.061*	

### 4-Methylquinolinium 4-chloro-2-nitrobenzoate (V) Atomic displacement parameters (Å^2^)

**Table:**

	*U*^11^	*U*^22^	*U*^33^	*U*^12^	*U*^13^	*U*^23^
Cl1	0.0431 (2)	0.0486 (2)	0.03629 (19)	−0.01563 (16)	−0.01541 (14)	0.01651 (14)
O1	0.0291 (4)	0.0417 (5)	0.0307 (4)	−0.0157 (4)	−0.0063 (3)	0.0105 (4)
O2	0.0291 (4)	0.0476 (5)	0.0321 (4)	−0.0169 (4)	−0.0112 (3)	0.0094 (4)
O3	0.0271 (5)	0.0596 (7)	0.0399 (5)	0.0006 (5)	−0.0017 (4)	0.0002 (5)
O4	0.0427 (6)	0.0587 (7)	0.0673 (7)	−0.0319 (5)	−0.0236 (5)	0.0105 (5)
N1	0.0237 (5)	0.0446 (6)	0.0295 (5)	−0.0139 (5)	−0.0045 (4)	0.0094 (4)
N2	0.0260 (5)	0.0278 (5)	0.0268 (5)	−0.0114 (4)	−0.0037 (4)	0.0016 (4)
C1	0.0229 (5)	0.0244 (5)	0.0231 (5)	−0.0086 (4)	−0.0033 (4)	−0.0019 (4)
C2	0.0213 (5)	0.0285 (5)	0.0254 (5)	−0.0085 (4)	−0.0039 (4)	−0.0018 (4)
C3	0.0275 (6)	0.0331 (6)	0.0257 (5)	−0.0127 (5)	−0.0026 (4)	0.0017 (4)
C4	0.0313 (6)	0.0283 (5)	0.0243 (5)	−0.0089 (5)	−0.0073 (4)	0.0013 (4)
C5	0.0240 (5)	0.0328 (6)	0.0303 (6)	−0.0094 (4)	−0.0079 (4)	0.0000 (5)
C6	0.0244 (5)	0.0298 (5)	0.0277 (5)	−0.0116 (4)	−0.0036 (4)	−0.0010 (4)
C7	0.0252 (5)	0.0262 (5)	0.0245 (5)	−0.0083 (4)	−0.0038 (4)	−0.0003 (4)
C8	0.0270 (6)	0.0286 (5)	0.0333 (6)	−0.0096 (4)	−0.0013 (4)	−0.0035 (5)
C9	0.0334 (6)	0.0371 (6)	0.0255 (5)	−0.0168 (5)	0.0003 (4)	−0.0042 (5)
C10	0.0305 (6)	0.0346 (6)	0.0272 (5)	−0.0189 (5)	−0.0057 (4)	0.0031 (5)
C11	0.0249 (5)	0.0275 (5)	0.0294 (5)	−0.0135 (4)	−0.0044 (4)	0.0007 (4)
C12	0.0295 (6)	0.0293 (6)	0.0450 (7)	−0.0105 (5)	−0.0086 (5)	0.0014 (5)
C13	0.0295 (6)	0.0317 (6)	0.0548 (8)	−0.0079 (5)	−0.0006 (5)	−0.0104 (6)
C14	0.0362 (7)	0.0434 (7)	0.0386 (7)	−0.0168 (6)	0.0054 (5)	−0.0141 (6)
C15	0.0345 (6)	0.0379 (6)	0.0278 (6)	−0.0173 (5)	−0.0010 (5)	−0.0035 (5)
C16	0.0246 (5)	0.0268 (5)	0.0270 (5)	−0.0131 (4)	−0.0027 (4)	−0.0016 (4)
C17	0.0429 (7)	0.0485 (8)	0.0309 (6)	−0.0212 (6)	−0.0104 (5)	0.0106 (6)

### 4-Methylquinolinium 4-chloro-2-nitrobenzoate (V) Geometric parameters (Å, º)

**Table:**

Cl1—C4	1.7291 (12)	C8—C9	1.3887 (17)
O1—C7	1.2804 (14)	C8—H8	0.9500
O2—C7	1.2313 (14)	C9—C10	1.3699 (18)
O3—N1	1.2127 (15)	C9—H9	0.9500
O4—N1	1.2220 (16)	C10—C11	1.4239 (16)
N1—C2	1.4780 (14)	C10—C17	1.4938 (17)
N2—C8	1.3156 (15)	C11—C16	1.4107 (16)
N2—C16	1.3652 (15)	C11—C12	1.4132 (17)
N2—H2	1.06 (2)	C12—C13	1.364 (2)
C1—C2	1.3936 (15)	C12—H12	0.9500
C1—C6	1.3948 (15)	C13—C14	1.405 (2)
C1—C7	1.5085 (15)	C13—H13	0.9500
C2—C3	1.3790 (16)	C14—C15	1.3601 (19)
C3—C4	1.3859 (17)	C14—H14	0.9500
C3—H3	0.9500	C15—C16	1.4062 (16)
C4—C5	1.3863 (17)	C15—H15	0.9500
C5—C6	1.3834 (16)	C17—H17A	0.9800
C5—H5	0.9500	C17—H17B	0.9800
C6—H6	0.9500	C17—H17C	0.9800
			
O3—N1—O4	125.32 (12)	C10—C9—C8	120.82 (11)
O3—N1—C2	117.47 (11)	C10—C9—H9	119.6
O4—N1—C2	117.09 (11)	C8—C9—H9	119.6
C8—N2—C16	120.67 (10)	C9—C10—C11	117.95 (11)
C8—N2—H2	120.5 (12)	C9—C10—C17	121.06 (11)
C16—N2—H2	118.8 (12)	C11—C10—C17	120.99 (11)
C2—C1—C6	116.89 (10)	C16—C11—C12	118.02 (11)
C2—C1—C7	122.38 (10)	C16—C11—C10	118.57 (10)
C6—C1—C7	120.58 (10)	C12—C11—C10	123.40 (11)
C3—C2—C1	123.52 (10)	C13—C12—C11	120.30 (12)
C3—C2—N1	115.15 (10)	C13—C12—H12	119.9
C1—C2—N1	121.33 (10)	C11—C12—H12	119.9
C2—C3—C4	117.23 (10)	C12—C13—C14	120.75 (12)
C2—C3—H3	121.4	C12—C13—H13	119.6
C4—C3—H3	121.4	C14—C13—H13	119.6
C3—C4—C5	121.84 (11)	C15—C14—C13	120.82 (12)
C3—C4—Cl1	118.83 (9)	C15—C14—H14	119.6
C5—C4—Cl1	119.33 (9)	C13—C14—H14	119.6
C6—C5—C4	118.97 (11)	C14—C15—C16	119.15 (12)
C6—C5—H5	120.5	C14—C15—H15	120.4
C4—C5—H5	120.5	C16—C15—H15	120.4
C5—C6—C1	121.49 (11)	N2—C16—C15	118.72 (11)
C5—C6—H6	119.3	N2—C16—C11	120.32 (10)
C1—C6—H6	119.3	C15—C16—C11	120.96 (11)
O2—C7—O1	126.25 (11)	C10—C17—H17A	109.5
O2—C7—C1	118.37 (10)	C10—C17—H17B	109.5
O1—C7—C1	115.33 (10)	H17A—C17—H17B	109.5
N2—C8—C9	121.66 (11)	C10—C17—H17C	109.5
N2—C8—H8	119.2	H17A—C17—H17C	109.5
C9—C8—H8	119.2	H17B—C17—H17C	109.5
			
C6—C1—C2—C3	−2.12 (17)	C16—N2—C8—C9	−0.01 (17)
C7—C1—C2—C3	173.58 (10)	N2—C8—C9—C10	−0.70 (18)
C6—C1—C2—N1	177.81 (10)	C8—C9—C10—C11	1.00 (17)
C7—C1—C2—N1	−6.48 (16)	C8—C9—C10—C17	−179.56 (11)
O3—N1—C2—C3	97.04 (13)	C9—C10—C11—C16	−0.65 (16)
O4—N1—C2—C3	−79.28 (14)	C17—C10—C11—C16	179.91 (10)
O3—N1—C2—C1	−82.90 (14)	C9—C10—C11—C12	178.67 (11)
O4—N1—C2—C1	100.78 (14)	C17—C10—C11—C12	−0.77 (18)
C1—C2—C3—C4	0.31 (18)	C16—C11—C12—C13	0.21 (17)
N1—C2—C3—C4	−179.63 (10)	C10—C11—C12—C13	−179.11 (11)
C2—C3—C4—C5	1.49 (18)	C11—C12—C13—C14	−0.6 (2)
C2—C3—C4—Cl1	−178.45 (9)	C12—C13—C14—C15	0.2 (2)
C3—C4—C5—C6	−1.37 (18)	C13—C14—C15—C16	0.49 (19)
Cl1—C4—C5—C6	178.58 (9)	C8—N2—C16—C15	−179.53 (11)
C4—C5—C6—C1	−0.58 (18)	C8—N2—C16—C11	0.35 (16)
C2—C1—C6—C5	2.23 (16)	C14—C15—C16—N2	179.02 (11)
C7—C1—C6—C5	−173.56 (10)	C14—C15—C16—C11	−0.86 (18)
C2—C1—C7—O2	−5.64 (16)	C12—C11—C16—N2	−179.37 (10)
C6—C1—C7—O2	169.91 (11)	C10—C11—C16—N2	−0.01 (16)
C2—C1—C7—O1	176.77 (10)	C12—C11—C16—C15	0.51 (16)
C6—C1—C7—O1	−7.68 (15)	C10—C11—C16—C15	179.86 (10)

### 4-Methylquinolinium 4-chloro-2-nitrobenzoate (V) Hydrogen-bond geometry (Å, º)

**Table:**

*D*—H···*A*	*D*—H	H···*A*	*D*···*A*	*D*—H···*A*
N2—H2···O1	1.06 (2)	1.50 (2)	2.5568 (13)	179 (4)
C8—H8···O2^i^	0.95	2.56	3.2779 (16)	132
C12—H12···O2^ii^	0.95	2.52	3.3391 (18)	144

Symmetry codes: (i) −*x*+1, −*y*, −*z*+1; (ii) −*x*, −*y*+1, −*z*+1.

### 4-Methylquinolinium 5-chloro-2-nitrobenzoate (VI) Crystal data

**Table:**

C_10_H_10_N^+^.C_7_H_3_ClNO_4_^−^	*F*(000) = 1424.00
*M**_r_* = 344.75	*D*_x_ = 1.502 Mg m^−^^3^
Monoclinic, *C*2/*c*	Mo *K*α radiation, λ = 0.71075 Å
*a* = 16.2625 (10) Å	Cell parameters from 24067 reflections
*b* = 7.5099 (4) Å	θ = 3.0–30.0°
*c* = 25.3105 (15) Å	µ = 0.28 mm^−^^1^
β = 99.4086 (19)°	*T* = 190 K
*V* = 3049.6 (3) Å^3^	Prism, colorless
*Z* = 8	0.30 × 0.21 × 0.12 mm

### 4-Methylquinolinium 5-chloro-2-nitrobenzoate (VI) Data collection

**Table:**

Rigaku R-AXIS RAPIDII diffractometer	3913 reflections with *I* > 2σ(*I*)
Detector resolution: 10.000 pixels mm^-1^	*R*_int_ = 0.022
ω scans	θ_max_ = 30.0°, θ_min_ = 3.0°
Absorption correction: numerical (NUMABS; Higashi, 1999)	*h* = −22→22
*T*_min_ = 0.916, *T*_max_ = 0.968	*k* = −10→10
29037 measured reflections	*l* = −35→35
4457 independent reflections	

### 4-Methylquinolinium 5-chloro-2-nitrobenzoate (VI) Refinement

**Table:**

Refinement on *F*^2^	Secondary atom site location: difference Fourier map
*R*[*F*^2^ > 2σ(*F*^2^)] = 0.036	Hydrogen site location: inferred from neighbouring sites
*wR*(*F*^2^) = 0.099	H atoms treated by a mixture of independent and constrained refinement
*S* = 1.05	*w* = 1/[σ^2^(*F*_o_^2^) + (0.0586*P*)^2^ + 1.3567*P*] where *P* = (*F*_o_^2^ + 2*F*_c_^2^)/3
4457 reflections	(Δ/σ)_max_ = 0.001
222 parameters	Δρ_max_ = 0.47 e Å^−^^3^
0 restraints	Δρ_min_ = −0.16 e Å^−^^3^
Primary atom site location: structure-invariant direct methods	

### 4-Methylquinolinium 5-chloro-2-nitrobenzoate (VI) Special details

**Table:**

Geometry. All esds (except the esd in the dihedral angle between two l.s. planes) are estimated using the full covariance matrix. The cell esds are taken into account individually in the estimation of esds in distances, angles and torsion angles; correlations between esds in cell parameters are only used when they are defined by crystal symmetry. An approximate (isotropic) treatment of cell esds is used for estimating esds involving l.s. planes.

### 4-Methylquinolinium 5-chloro-2-nitrobenzoate (VI) Fractional atomic coordinates and isotropic or equivalent isotropic displacement parameters (Å^2^)

**Table:**

	*x*	*y*	*z*	*U*_iso_*/*U*_eq_	
Cl1	0.35497 (2)	0.25440 (3)	0.21927 (2)	0.03174 (9)	
O1	0.58343 (5)	0.52232 (13)	0.37347 (3)	0.0379 (2)	
O2	0.51224 (5)	0.73265 (12)	0.40930 (3)	0.03410 (19)	
O3	0.50836 (5)	1.00336 (12)	0.32498 (4)	0.0393 (2)	
O4	0.38124 (6)	1.04110 (12)	0.33823 (3)	0.03630 (19)	
N1	0.43689 (6)	0.95186 (12)	0.32389 (3)	0.02602 (17)	
N2	0.70950 (5)	0.49554 (12)	0.44591 (3)	0.02496 (17)	
C1	0.45930 (6)	0.62497 (13)	0.32235 (4)	0.02159 (18)	
C2	0.41499 (6)	0.77444 (13)	0.30138 (4)	0.02245 (18)	
C3	0.35187 (7)	0.76754 (14)	0.25743 (4)	0.0276 (2)	
H3	0.322449	0.872401	0.244702	0.033*	
C4	0.33229 (6)	0.60562 (15)	0.23234 (4)	0.0279 (2)	
H4	0.289265	0.597431	0.202146	0.033*	
C5	0.37668 (6)	0.45573 (13)	0.25212 (4)	0.02356 (18)	
C6	0.43909 (6)	0.46287 (13)	0.29667 (4)	0.02320 (18)	
H6	0.467928	0.357532	0.309587	0.028*	
C7	0.52359 (6)	0.63089 (13)	0.37316 (4)	0.02337 (18)	
C8	0.77132 (6)	0.42632 (14)	0.42468 (4)	0.0276 (2)	
H8	0.763497	0.406693	0.387111	0.033*	
C9	0.84757 (6)	0.38107 (14)	0.45538 (4)	0.0267 (2)	
H9	0.890820	0.332639	0.438694	0.032*	
C10	0.86007 (6)	0.40684 (13)	0.51013 (4)	0.02478 (19)	
C11	0.79365 (6)	0.48165 (13)	0.53362 (4)	0.02323 (18)	
C12	0.79902 (7)	0.51180 (16)	0.58948 (4)	0.0313 (2)	
H12	0.849207	0.485426	0.613072	0.038*	
C13	0.73200 (8)	0.57880 (18)	0.60938 (5)	0.0362 (2)	
H13	0.735942	0.596465	0.646879	0.043*	
C14	0.65709 (7)	0.62213 (17)	0.57515 (5)	0.0335 (2)	
H14	0.611455	0.669217	0.589773	0.040*	
C15	0.64983 (6)	0.59662 (15)	0.52088 (4)	0.0283 (2)	
H15	0.599673	0.626737	0.497774	0.034*	
C16	0.71795 (6)	0.52500 (13)	0.49994 (4)	0.02273 (18)	
C17	0.94142 (7)	0.35605 (17)	0.54355 (5)	0.0340 (2)	
H17A	0.967677	0.462034	0.561585	0.051*	
H17B	0.978265	0.304870	0.520528	0.051*	
H17C	0.931537	0.268016	0.570401	0.051*	
H2	0.6559 (13)	0.524 (3)	0.4196 (9)	0.074 (6)*	

### 4-Methylquinolinium 5-chloro-2-nitrobenzoate (VI) Atomic displacement parameters (Å^2^)

**Table:**

	*U*^11^	*U*^22^	*U*^33^	*U*^12^	*U*^13^	*U*^23^
Cl1	0.03751 (15)	0.02625 (13)	0.03075 (14)	−0.00503 (9)	0.00350 (11)	−0.00484 (9)
O1	0.0330 (4)	0.0476 (5)	0.0287 (4)	0.0189 (4)	−0.0077 (3)	−0.0084 (3)
O2	0.0337 (4)	0.0406 (5)	0.0259 (4)	0.0105 (3)	−0.0015 (3)	−0.0072 (3)
O3	0.0335 (4)	0.0350 (4)	0.0470 (5)	−0.0106 (3)	−0.0008 (4)	−0.0006 (4)
O4	0.0455 (5)	0.0292 (4)	0.0349 (4)	0.0063 (3)	0.0084 (4)	−0.0037 (3)
N1	0.0308 (4)	0.0231 (4)	0.0224 (4)	−0.0004 (3)	−0.0007 (3)	0.0023 (3)
N2	0.0237 (4)	0.0265 (4)	0.0234 (4)	0.0025 (3)	0.0004 (3)	0.0026 (3)
C1	0.0187 (4)	0.0252 (4)	0.0205 (4)	0.0016 (3)	0.0021 (3)	0.0008 (3)
C2	0.0220 (4)	0.0225 (4)	0.0222 (4)	0.0001 (3)	0.0018 (3)	0.0002 (3)
C3	0.0257 (5)	0.0259 (5)	0.0282 (5)	0.0033 (3)	−0.0041 (4)	0.0019 (4)
C4	0.0249 (4)	0.0299 (5)	0.0261 (4)	0.0000 (4)	−0.0044 (4)	−0.0005 (4)
C5	0.0233 (4)	0.0238 (4)	0.0237 (4)	−0.0025 (3)	0.0043 (3)	−0.0021 (3)
C6	0.0218 (4)	0.0238 (4)	0.0240 (4)	0.0022 (3)	0.0038 (3)	0.0015 (3)
C7	0.0220 (4)	0.0262 (4)	0.0208 (4)	0.0016 (3)	0.0001 (3)	0.0020 (3)
C8	0.0283 (5)	0.0294 (5)	0.0248 (4)	0.0018 (4)	0.0038 (4)	0.0015 (4)
C9	0.0234 (4)	0.0270 (5)	0.0300 (5)	0.0023 (3)	0.0049 (4)	0.0009 (4)
C10	0.0213 (4)	0.0212 (4)	0.0303 (5)	−0.0004 (3)	−0.0004 (3)	0.0018 (4)
C11	0.0220 (4)	0.0216 (4)	0.0247 (4)	−0.0012 (3)	−0.0005 (3)	0.0020 (3)
C12	0.0300 (5)	0.0361 (6)	0.0252 (5)	−0.0005 (4)	−0.0028 (4)	−0.0005 (4)
C13	0.0389 (6)	0.0431 (6)	0.0258 (5)	0.0008 (5)	0.0026 (4)	−0.0053 (5)
C14	0.0321 (5)	0.0360 (6)	0.0333 (5)	0.0040 (4)	0.0084 (4)	−0.0044 (4)
C15	0.0247 (4)	0.0290 (5)	0.0307 (5)	0.0040 (4)	0.0029 (4)	0.0001 (4)
C16	0.0226 (4)	0.0208 (4)	0.0238 (4)	0.0002 (3)	0.0008 (3)	0.0021 (3)
C17	0.0226 (5)	0.0376 (6)	0.0387 (6)	0.0040 (4)	−0.0042 (4)	0.0001 (5)

### 4-Methylquinolinium 5-chloro-2-nitrobenzoate (VI) Geometric parameters (Å, º)

**Table:**

Cl1—C5	1.7338 (10)	C8—C9	1.3935 (14)
O1—C7	1.2686 (12)	C8—H8	0.9500
O2—C7	1.2288 (13)	C9—C10	1.3811 (15)
O3—N1	1.2211 (12)	C9—H9	0.9500
O4—N1	1.2277 (12)	C10—C11	1.4303 (14)
N1—C2	1.4698 (13)	C10—C17	1.4985 (14)
N2—C8	1.3215 (13)	C11—C16	1.4159 (13)
N2—C16	1.3700 (13)	C11—C12	1.4204 (14)
N2—H2	1.03 (2)	C12—C13	1.3691 (17)
C1—C2	1.3913 (13)	C12—H12	0.9500
C1—C6	1.3938 (14)	C13—C14	1.4128 (17)
C1—C7	1.5197 (13)	C13—H13	0.9500
C2—C3	1.3858 (14)	C14—C15	1.3726 (16)
C3—C4	1.3847 (15)	C14—H14	0.9500
C3—H3	0.9500	C15—C16	1.4105 (14)
C4—C5	1.3859 (14)	C15—H15	0.9500
C4—H4	0.9500	C17—H17A	0.9800
C5—C6	1.3893 (14)	C17—H17B	0.9800
C6—H6	0.9500	C17—H17C	0.9800
			
O3—N1—O4	124.43 (10)	C10—C9—C8	119.80 (9)
O3—N1—C2	117.73 (9)	C10—C9—H9	120.1
O4—N1—C2	117.77 (9)	C8—C9—H9	120.1
C8—N2—C16	120.70 (9)	C9—C10—C11	118.34 (9)
C8—N2—H2	116.0 (12)	C9—C10—C17	120.16 (9)
C16—N2—H2	123.3 (12)	C11—C10—C17	121.50 (9)
C2—C1—C6	117.31 (8)	C16—C11—C12	117.94 (9)
C2—C1—C7	122.75 (9)	C16—C11—C10	118.79 (9)
C6—C1—C7	119.82 (8)	C12—C11—C10	123.27 (9)
C3—C2—C1	123.05 (9)	C13—C12—C11	120.11 (10)
C3—C2—N1	116.47 (9)	C13—C12—H12	119.9
C1—C2—N1	120.40 (8)	C11—C12—H12	119.9
C4—C3—C2	119.06 (9)	C12—C13—C14	121.17 (10)
C4—C3—H3	120.5	C12—C13—H13	119.4
C2—C3—H3	120.5	C14—C13—H13	119.4
C3—C4—C5	118.71 (9)	C15—C14—C13	120.40 (10)
C3—C4—H4	120.6	C15—C14—H14	119.8
C5—C4—H4	120.6	C13—C14—H14	119.8
C4—C5—C6	122.02 (9)	C14—C15—C16	119.00 (10)
C4—C5—Cl1	118.81 (8)	C14—C15—H15	120.5
C6—C5—Cl1	119.16 (8)	C16—C15—H15	120.5
C5—C6—C1	119.83 (9)	N2—C16—C15	118.68 (9)
C5—C6—H6	120.1	N2—C16—C11	119.95 (9)
C1—C6—H6	120.1	C15—C16—C11	121.37 (9)
O2—C7—O1	127.13 (9)	C10—C17—H17A	109.5
O2—C7—C1	118.80 (9)	C10—C17—H17B	109.5
O1—C7—C1	114.03 (8)	H17A—C17—H17B	109.5
N2—C8—C9	122.41 (10)	C10—C17—H17C	109.5
N2—C8—H8	118.8	H17A—C17—H17C	109.5
C9—C8—H8	118.8	H17B—C17—H17C	109.5
			
C6—C1—C2—C3	1.16 (15)	C16—N2—C8—C9	−0.41 (16)
C7—C1—C2—C3	−174.95 (9)	N2—C8—C9—C10	0.62 (17)
C6—C1—C2—N1	−175.55 (8)	C8—C9—C10—C11	−0.59 (15)
C7—C1—C2—N1	8.33 (14)	C8—C9—C10—C17	179.10 (10)
O3—N1—C2—C3	−120.32 (11)	C9—C10—C11—C16	0.39 (14)
O4—N1—C2—C3	56.69 (13)	C17—C10—C11—C16	−179.29 (10)
O3—N1—C2—C1	56.60 (13)	C9—C10—C11—C12	179.10 (10)
O4—N1—C2—C1	−126.39 (10)	C17—C10—C11—C12	−0.58 (16)
C1—C2—C3—C4	−1.07 (16)	C16—C11—C12—C13	0.76 (16)
N1—C2—C3—C4	175.77 (9)	C10—C11—C12—C13	−177.96 (11)
C2—C3—C4—C5	−0.02 (16)	C11—C12—C13—C14	−1.11 (19)
C3—C4—C5—C6	0.97 (16)	C12—C13—C14—C15	0.4 (2)
C3—C4—C5—Cl1	−177.60 (8)	C13—C14—C15—C16	0.59 (18)
C4—C5—C6—C1	−0.86 (15)	C8—N2—C16—C15	−179.05 (10)
Cl1—C5—C6—C1	177.70 (7)	C8—N2—C16—C11	0.20 (15)
C2—C1—C6—C5	−0.20 (14)	C14—C15—C16—N2	178.31 (10)
C7—C1—C6—C5	176.04 (9)	C14—C15—C16—C11	−0.93 (16)
C2—C1—C7—O2	34.14 (15)	C12—C11—C16—N2	−178.97 (9)
C6—C1—C7—O2	−141.88 (10)	C10—C11—C16—N2	−0.19 (14)
C2—C1—C7—O1	−147.73 (10)	C12—C11—C16—C15	0.25 (15)
C6—C1—C7—O1	36.25 (13)	C10—C11—C16—C15	179.04 (9)

### 4-Methylquinolinium 5-chloro-2-nitrobenzoate (VI) Hydrogen-bond geometry (Å, º)

**Table:**

*D*—H···*A*	*D*—H	H···*A*	*D*···*A*	*D*—H···*A*
N2—H2···O1	1.03 (2)	1.52 (2)	2.5252 (11)	165 (2)
C9—H9···O2^i^	0.95	2.34	3.2856 (13)	171
C12—H12···O3^ii^	0.95	2.58	3.5065 (14)	166
C15—H15···O2	0.95	2.57	3.4583 (13)	155
C17—H17*A*···O2^ii^	0.98	2.41	3.3524 (16)	160

Symmetry codes: (i) *x*+1/2, *y*−1/2, *z*; (ii) −*x*+3/2, −*y*+3/2, −*z*+1.
